# Ghrelin Regulates Expression of the Transcription Factor Pax6 in Hypoxic Brain Progenitor Cells and Neurons

**DOI:** 10.3390/cells11050782

**Published:** 2022-02-23

**Authors:** Irina I. Stoyanova, Andrii Klymenko, Jeannette Willms, Thorsten R. Doeppner, Anton B. Tonchev, David Lutz

**Affiliations:** 1 Department of Anatomy and Cell Biology, Faculty of Medicine, Research Institute of the Medical University, 9002 Varna, Bulgaria; anton.tonchev@mu-varna.bg; 2 Department of Neuroanatomy and Molecular Brain Research, Ruhr University Bochum, 44801 Bochum, Germany; andrii.klymenko@rub.de (A.K.); jeannette.willms@ruhr-uni-bochum.de (J.W.); 3 Department of Neurology, University Medical Center Goettingen, 37075 Goettingen, Germany; thorsten.doeppner@med.uni-goettingen.de; 4 Research Institute for Health Sciences and Technologies (SABITA), Medipol University, Istanbul 34810, Turkey

**Keywords:** GHSR1, ghrelin, hypoxia, neurogenesis, transcription factors, progenitor cells

## Abstract

The nature of brain impairment after hypoxia is complex and recovery harnesses different mechanisms, including neuroprotection and neurogenesis. Experimental evidence suggests that hypoxia may trigger neurogenesis postnatally by influencing the expression of a variety of transcription factors. However, the existing data are controversial. As a proof-of-principle, we subjected cultured cerebral cortex neurons, cerebellar granule neurons and organotypic cerebral cortex slices from rat brains to hypoxia and treated these cultures with the hormone ghrelin, which is well-known for its neuroprotective functions. We found that hypoxia elevated the expression levels and stimulated nuclear translocation of ghrelin’s receptor GHSR1 in the cultured neurons and the acute organotypic slices, whereas ghrelin treatment reduced the receptor expression to normoxic levels. GHSR1 expression was also increased in cerebral cortex neurons of mice with induced experimental stroke. Additional quantitative analyses of immunostainings for neuronal proliferation and differentiation markers revealed that hypoxia stimulated the proliferation of neuronal progenitors, whereas ghrelin application during the phase of recovery from hypoxia counteracted these effects. At the mechanistic level, we provide a link between the described post-ischemic phenomena and the expression of the transcription factor Pax6, an important regulator of neural progenitor cell fate. In contrast to the neurogenic niches in the brain where hypoxia is known to increase Pax6 expression, the levels of the transcription factor in cultured hypoxic cerebral cortex cells were downregulated. Moreover, the application of ghrelin to hypoxic neurons normalised the expression levels of these factors. Our findings suggest that ghrelin stimulates neurogenic factors for the protection of neurons in a GHSR1-dependent manner in non-neurogenic brain areas such as the cerebral cortex after exposure to hypoxia.

## 1. Introduction

Stroke is a medical condition of impeded blood supply to the brain and oxygen shortage (hypoxia). The recovery from hypoxia requires intra- and extracellular processes recapitulating nervous system development such as extracellular matrix reorganisation, neurogenesis, and stimulation of neuronal plasticity. A prolonged hypoxia state may result in ischemia followed by neuronal death and reduction of neuronal density [1]. Interestingly, hypoxia can also trigger neurogenesis within the surrounding tissue [2,3] prenatally as well as postpartum [4,5]. Neurogenesis in the adult brain is essential and occurs during the entire postnatal life. It is believed to be restricted to the subventricular zone and the subgranular zone of the dentate gyrus, though it has been observed in some other areas of the mammalian brain, e.g., the subcallosal zone [6], the striatum [7], the amygdala [8], and also the neocortex [9], albeit at considerably lower levels. The adult neurogenesis goes through the same consecutive stages as the embryonic one does and yields post-mitotic functionally integrated new neurons [10].

Exposure to hypoxia during the early postnatal period activates generation of Tbr1-positive spiny pyramidal neurons in vitro as well as ex vivo [11]. In vitro, some of the early neuronal stem cells can acquire multipotency and undergo long-term self-renewal upon exposure to hormones and growth- and transcription factors [12].

*Pax6* is a member of the paired-box and homeobox-containing gene family (PAX) of transcription factors and its early expressed protein Pax6 is a key transcription factor in the generation of neuronal lineages during the development of the central nervous system [13,14,15]. Moreover, *Pax6* is highly conserved between species: there is no difference between the amino acid sequence of the human and mouse Pax6, thus pointing to a pivotal role in brain development [16]. Indeed, *Pax6* regulates corticogenesis, numbers and arrangement of cortical cells in layers as well as the ratio of excitatory and inhibitory neurons [5]; these effects are dose-dependent [17]. *Pax6* is considered a neurogenic fate determinant directing astrocyte-to-neuron conversion during adult neurogenesis.

Unlike the neurogenic niches, where hypoxia increases Pax6 expression, the levels in the neocortex are downregulated [2]. Does this decreased neocortical expression of Pax6 act neuroprotectively on cortical neurons? Could Pax6 upregulation influence neurogenesis, and thus, benefit recovery from hypoxia? As a proof-of-principle, we subjected dissociated cerebral cortex neurons, cerebellar granule neurons and acute cerebral cortex slices to hypoxia and treated them with the hormone ghrelin, which is well-known to act neuroprotectively against oxidative stress in vivo [18] and in vitro [19]. We then examined the effect of hypoxia on the expression levels of ghrelin’s receptor GHSR1 (growth hormone secretagogue receptor 1) in cultured neurons and acute organotypic slices. We also confirmed that the expression of GHSR1 is upregulated in the cerebral cortex of mice with transitional middle cerebral artery occlusion used as an in vivo stroke model. Furthermore, we subjected hypoxic cultures of mature cerebral cortex neurons that had been treated with ghrelin to immunostaining for Pax6, Ki67 (neuronal proliferation and differentiation marker), or NeuN (neuronal nuclear antigen) aiming at analysing the changes in the ratio of cells positive for these markers upon hypoxia vs. normoxic conditions.

## 2. Materials and Methods

### 2.1. Animals Used for Primary Cell Cultures

All animal experiments were conducted according to German law and approved by the corresponding committee on animal use, being conformed to the guidelines set by the European Union. Wistar rats were bred and maintained at 22 °C on a 12 h light/dark cycle with *ad libitum* food and water access in the Animal Facility of the Medical Faculty at the Ruhr University Bochum, Germany. For in vitro experiments, a mixed population of female and male offspring was used. The manuscript was prepared following the ARRIVE guidelines for animal research [20].

### 2.2. Animals Used for Experimental Ischemia In Vivo

Permission for the animal experiments was received by the Lower Saxony State Office for Consumer Protection and Food Safety of the City Oldenburg (Niedersächsisches Landesamt für Verbraucherschutz und Lebesmittelsicherheit (LAVES)/Oldenburg, contract No 33.9-42502-04-11/0622). Six-month-old C57Bl6 mice were subjected to transient focal cerebral ischemia as described [21]. Briefly, animals were anaesthetised by inhalation of 0.8–1.5% isoflurane, 30% O_2_, and remainder N_2_O. The rectal temperature was maintained at 36.5–37.0 °C by employing a feedback-controlled heating system under continuous blood flow monitoring using a laser Doppler flow (LDF) system (Perimed, Järfälla, Sweden). Occlusion of the middle cerebral artery was achieved with a 7–0 silicon coated nylon monofilament (tip diameter of 180 µm; Doccol, Sharon, MA, USA) that was withdrawn after 45 min to entail transient cerebral ischemia. LDF recordings were continued for additional 15 min to monitor and verify the appropriate reperfusion of the brain. Animals were sacrificed on day 28 after the induction of stroke and subjected to immunostaining for detection of the ghrelin receptor GHSR1 as described in Section 2.6.

### 2.3. Dissociated Cell Cultures

Cerebral cortex neurons were obtained from newborn Wistar rats, from six plating procedures, five pups (from the same mother) per plating. In brief, neonates were anaesthetised by isoflurane inhalation and decapitated. Brains were isolated, the cerebral cortices were dissected and placed in Neurobasal medium (Thermo Fisher Scientific, Waltham, MA, USA). After removal of the meninges, the cerebral cortices were collected in a chemically defined R12 culture medium [22] with trypsin for chemical dissociation. Thereafter, 150 µL of soybean trypsin inhibitor and 125 µL of DNAse I (20.000 units, Thermo Fisher Scientific) were added, followed by trituration for mechanical dissociation of the cells. The suspension was centrifuged at 1200 rpm at 4 °C for 5 min. Cells were plated on glass coverslips at a density of approximately 3000 cells/mm^2^. The glass coverslips were pre-coated with 20 mg/mL poly-L-lysine (Merck, Darmstadt, Germany) to enhance cell adhesion. Cells were allowed to settle at 37 °C and 5% CO_2_ for 2 h (incubation in R12 medium, optimised with 50 ng/mL nerve growth factor (Thermo Fisher Scientific)). The medium was serum-free to suppress glial cell proliferation, thus keeping glial cell numbers lower than 5% [22]. The medium was renewed twice a week. The cultures were stored in an incubator under standard conditions of 37 °C, 100% humidity and 5% CO_2_ for a period of one or three weeks prior to hypoxia.

Cerebellar granule neurons were obtained from a mixed population of 4-day-old female and male Wistar rats. Briefly, the animals were anaesthetised by isoflurane inhalation and decapitated. Brains were isolated, the cerebella were dissected and placed in Neurobasal medium (Thermo Fisher Scientific). After removal of the meninges, the cerebella were incubated in 0.025% trypsin (Merck) in Hank’s balanced salt solution (HBSS, Thermo Fisher Scientific) at 37 °C for 30 min. The tissue was then incubated in HBSS containing 1% BSA (Merck) and 1% w/v trypsin inhibitor (cat.# T-6522, Merck) at 37 °C for 5 min. After washing in HBSS, the tissue was triturated with a pipette tip, and the dissociated neurons were cultured in neurobasal medium supplemented with 2% B-27 (Thermo Fisher Scientific), 0.5 mM L-glutamine (Thermo Fisher Scientific), 100 units/mL penicillin (Thermo Fisher Scientific), and 100 μg/mL streptomycin (Thermo Fisher Scientific) at a density of 1.7 × 10^4^ cells per well of a 24-well plate coated with poly-L-lysine (Merck). Cerebellar granule neurons were cultured for 24 h prior to hypoxia induction.

### 2.4. Acute Cerebral Cortex Slices

Acute cerebral cortex slices were obtained from a mixed population of 4-day-old female and male Wistar rats. The animals were anaesthetised by isoflurane inhalation and decapitated. Brains were isolated, the brain hemispheres were dissected in DMEM medium (Merck) and sliced at a thickness of 400 μm perpendicularly to their longitudinal axis using the McIlwain tissue chopper (Plano, Wetzlar, Germany). Slices were placed onto Millicell membrane inserts (Merck) and transferred into six-well plates with 1 mL of nutrition medium per well (25% heat-inactivated horse serum, 25% HBSS, 50% DMEM, 2 mM glutamine, pH 7.2). Slices were maintained under standard conditions of 37 °C, 100% humidity and 5% CO_2_ for a period of 24 h prior to hypoxia.

### 2.5. Induction of Hypoxia and Treatment with Ghrelin 

One-third of the cultured cells/acute slices were kept under normoxic conditions (37 °C, 100% humidity and 5% CO_2_) and the rest of the cultures were exposed to hypoxia for 6 h. Hypoxia was achieved by subjecting the cultures and slices to air evacuation [23], which took ~3–4 s. The induction of hypoxia begins with lowering the partial oxygen pressure (pO_2_) from ~160 mmHg to less than 25 mmHg within 30 min. This process was visually inspected with an oxygen indicator. The specimens were air-evacuated and sealed in a plastic bag, and then incubated at 37 °C and in a humidified atmosphere for 6 h (for further details see also [23]). After six hours of hypoxia, the cultures and slices were maintained under normoxic conditions (37 °C, 100% humidity and 5% CO_2_) for 24 h. Thereby, half of these cultures/slices were supplemented with human ghrelin peptide (cat.# ab199421, Abcam, Cambridge, UK) for the duration of 24 h (final concentration of 0.5 µM as described elsewhere [24,25,26,27]). The other half of the cultures were kept in a plain medium, also for 24 h, and used as a control.

### 2.6. Immunostaining

Immunostaining was performed on 36 independent cultures, with equal cell density for each experimental condition (normoxia, hypoxia, hypoxia + ghrelin). Primary cell cultures and acute slices were fixed in 4% paraformaldehyde/phosphate buffered saline pH 7.4 (PBS, Merck) at room temperature for 20 min, washed in PBS for 5 min, immersed in 1% bovine serum albumin (in PBS containing 0.01% Triton-X) for 20 min and immunostained for calretinin, GHSR1, Pax6, Ki67, GFAP, and NeuN using the following primary antibodies (dilutions in PBS are indicated): mouse anti-GHSR1 (cat.# sc-374515, RRID:AB_10987651, Santa Cruz Biotechnology, Dallas, TX, USA, dilution 1:1000), rabbit anti-calretinin (cat.# CR 7697, RRID:AB_2619710, Swant, Burgdorf, Switzerland, dilution 1:1000), rabbit anti-Pax6 (cat.# PRB-278P, RRID:AB_291612, Covance, Princeton, NJ, USA, dilution 1:500) or mouse monoclonal anti-Pax6 (cat.# MA1-109, RRID:AB_2536820, Thermo Fisher Scientific), rabbit anti-Ki67 (cat.# VP-RM04, RRID:AB_2336545, Vector Laboratories, Burlingame, CA, USA, dilution 1:200), goat anti-GFAP (cat.# ab53554, RRID:AB_880202, Abcam, dilution 1:750), and guinea-pig anti-NeuN (cat.# ABN90, RRID:AB_11205592, Millipore, Burlington, MA, USA, dilution 1:2000). In the case of double immunostaining, the specimens were incubated sequentially in each of the primary antibodies at 4 °C for 10 h each. Following several washes in PBS, the specimens were incubated in the appropriate secondary antibodies (IgG conjugated to fluorochromes): goat anti-rabbit Alexa 488 nm (cat.# A-11008, RRID:AB_143165, Thermo Fisher Scientific, diluted 1:1000), goat anti-mouse 594 nm (cat.# A-11031, RRID:AB_144696, Thermo Fisher Scientific, diluted 1:1000), goat anti-guinea pig 594 nm (cat.# 106-585-003, RRID:AB_2337442 Jackson ImmunoResearch Labs, West Grove, PA, USA, diluted 1:500), donkey anti-goat Alexa 488 nm (cat.# A32814, RRID:AB_2762838, Thermo Fisher Scientific, diluted 1:500), and donkey anti-rabbit Alexa 594 nm (cat.# A32754, RRID:AB_2762827, Thermo Fisher Scientific, dilution 1:500) in a dark chamber at room temperature for 2 h. The samples were counterstained with the fluorescent dye 4′,6-diamidino-2-phenylindole DAPI (cat.# 10236276001, Merck, dilution 1:1000) at room temperature for 15 min. After several washes in PBS, specimens were rinsed in distilled water, embedded in fluorescent mounting medium (Carl Roth, Karlsruhe, Germany) on coverslips. For transmitted light microscopy, the incubation in primary mouse anti-GHSR1 antibody was followed by treatment with biotinylated secondary antibodies (donkey anti-mouse IgG, dilution 1:500, Jackson ImmunoResearch Labs) at room temperature for 2 h. After rinsing, the brain slices were incubated for 1 h in the Vectastain ABC-HRP Kit (6.25 µL/mL of each compound in PBS, cat.# PK-4002, Vector Laboratories) following the manufacturer’s instructions. The peroxidase activity was visualised with the SG substrate kit (cat.# SK-4700, Vector Laboratories) in PBS for 5 min, at room temperature. Finally, the specimens were rinsed in PBS and mounted on coverslips.

To test the specificity of the GHSR1 antibody, we conducted two control staining procedures as follows. Prior to staining, we incubated the murine primary GHSR1 antibody with murine pituitary/hypothalamus tissue homogenate to bind the antibody with detergent-solubilised GHSR1. In particular, tissue of the pituitary gland and hypothalamus from an 11–month–old male mouse was freshly isolated and homogenised in RIPA buffer (150 mM sodium chloride, 50 mM tris-HCl, 1% Nonidet P–40, 0.5% sodium deoxycholate, pH 8.0). The homogenate was then centrifuged at 13,000 rpm (room temperature) for 10 min and the supernatant was used for further incubation steps. Approximately 120 µg of the supernatant protein were mixed with 4 ng of the GHRS1 antibody at room temperature for 30 min. This mixture was applied to fixed cerebral cortex cells at 4 °C for 10 h, followed by the standard steps of immunocytochemistry as described above. For the second control staining procedure, we applied only the Alexa 594—conjugated secondary antibody (without the primary GHSR1 antibody) to the cells (using a dilution of 1:1000) for 2 h. In both cases, the GHSR1 fluorescence signal was barely detectable with the set-up of the spinning disk microscope as described in the imaging acquisition section.

### 2.7. Homogenate Preparation for Dot Blot Analysis

Cerebral cortex slices (described above) were homogenised in lysis buffer containing 100 mM Tris-HCl, pH 7.4 (Carl Roth), 12 mM magnesium acetate tetrahydrate (Merck), and 6 M urea (Sigma-Aldrich, St. Louis, MO, USA) under several freezing-refreezing rounds in liquid nitrogen. The total protein amount was measured on the Genova Nano micro-volume Life Science Spectrophotometer (Jenway, Staffordshire, UK) using a direct UV light detection set-up following the manufacturer’s instructions. For each condition, 20 µg total protein per dot were dropped onto a nitrocellulose membrane (cat.# 10600015, 0.2 µm, GE Healthcare, Chicago, IL, USA). The membrane was rinsed in Vilber washing buffer (cat.# PU4000500, Vilber, Collégien, France) at room temperature for 30 min and then incubated with a murine antibody recognising GHSR1 (cat.# sc-374515, RRID:AB_10987651, Santa Cruz Biotechnology, dilution 1:1000, incubation overnight) or mouse β -actin antibody (cat.# A5441, RRID:AB_476744, Sigma-Aldrich, dilution 1:10,000, incubation for 1 hour) which were diluted in the Vilber Purity^TM^ anti-mouse HRP reagent (cat.# PU4200100, Vilber) following the manufacturer’s instructions. The membranes were then incubated in the Vilber Purecl^TM^ Dura substrate (cat.# PU4400125, Vilber) according to the manufacturer’s instructions and subjected to chemiluminescence detection using the Vilber Fusion FX Imager (Vilber). The duration of the exposure for the GHSR1 and β -actin signal were 10 s and 20 s, respectively. For quantification of chemiluminescent signals, the ImageJ2 software (version 2.3.0/1.53f, Fiji) was used. The membranes were then stained with Ponceau S solution (cat.# P7170-1L, Sigma-Aldrich) for 4 min, washed in distilled water for 15 min and air-dried.

### 2.8. Image Acquisition

After immunostaining, the specimens were subjected to fluorescence microscopy using a confocal fluorescence spinning disc microscope (Nikon, Minato, Tokyo, Japan) equipped with the PCO Edge 5.5 sCMOS camera (noise: 1.4 electrons, resolution: 5.5 megapixel, dynamic range: 22,000:1, speed: 100 fps, stabilised by Peltier cooling, Visitron Systems, Puchheim, Germany) and the VS-LMS Laser-Merge-System for CSU-X1 and 2D FRAP Option (Visitron Systems). For acquisition of images, 405 nm (3.54 mW), 488 nm (3.85 mW) and 561 nm (4.11 mW) laser wave lengths were used. Images were taken using a 40× air-magnification objective (ELWD 40×/0.6 air s plan fluor, OFN22, DIC, N1, MRH08430) or a 60× water-magnification immersion objective (60×a/1.20 WI plan Apo vc, Nikon, OFN25, DIC, N2, MRD07602). The software used for the acquisition of images was VisiView (Version 4.4.018, 16 December 2019, Visitron Systems, license # 1434). All exposure times were set to be 1000 ms with binning 2, offset 0/0, gain 0, and a non-implemented digitiser. The images were further processed and analysed with the ImageJ2 software (version 2.3.0/1.53f, Fiji). Only uncropped original images are provided in the figures.

Transmitted light microscopic images were captured using a fully motorised wide-field microscope Zeiss AxioImager Z.2 (Carl Zeiss, Jena, Germany) with an AxioCam Mrm rev.3 monochrome CCD camera (Carl Zeiss), and AxioVision v.4.9 software through an EC Plan-Neofluar objective 5×. Shading from the irregular illumination field was corrected during the acquisition via the camera’s built-in shading correction. The images were savedin the zvi-format (Carl Zeiss) of the AxioVision software, stitched together, and exported to an 8 bit TIFF format for further processing and analysis with the ImageJ2 software (version 2.3.0/1.53f, Fiji).

### 2.9. Immunogold Transmission Electron Microscopy

Formaldehyde-fixed cultures (described above) were incubated with 1% bovine serum albumin in PBS at room temperature for 30 min, followed by an incubation with the mouse monoclonal antibody against GHSR1 (diluted 1:1000 in PBS) at 4 °C for 72 h. Specimens were washed in PBS three times (20 min for each round) and incubated with a donkey polyclonal antibody coupled to 10 nm gold (cat.# ab39593, Abcam, RRID:AB_954429, dilution 1:100 in PBS) at room temperature for 1 hour. After intensive rinsing in PBS (five rounds, 5 min for each round), sections were fixed with 1% glutaraldehyde in PBS at room temperature for 10 mins. Specimens were then incubated in 0.8% NaCl and 8% glucose at room temperature for 30 min and chilled on ice for a further incubation in an aqueous mixture of 2% OsO_4_ and 1% potassium hexacyanoferrate (III) (cat.# 31251, Sigma-Aldrich) for 2 h. The osmicated sections were washed in an ice-chilled aqueous mixture of 0.8% NaCl and 8% glucose for 30 min and dehydrated in 70% ethanol (2 × 15 min), 90% ethanol (2 × 15 min), 95% ethanol (2 × 15 min), 100% ethanol (2 × 30 min), and pure propylene oxide (2 × 20 min) on ice. Sections were then incubated in a propylene oxide-Araldite mixture (1:1) containing 3% accelerator on ice for 2 h and then at room temperature overnight. Sections were then transferred into Araldite containing 2% accelerator for 1 hour (at room temperature) and then the Araldite mixture was refreshed for the final embedding. The embedded sections were cured at 65°C overnight. Semithin sections (500 nm) were cut on an ultramicrotome (Ultracut R, Leica, Wetzlar, Germany), stained with 1% toluidine blue in borax (4 min) and inspected under the Leica DME light microscope (10× and 40× objectives). Ultrathin sections (55 nm) were then cut, mounted on 100-meshed Nickel grids (Ted Pella, Redding, CA, USA) and stained with an aqueous solution of 4% uranyl acetate (cat. # E22400, Science Services, München, Germany) at room temperature for 20 min. The sections were rinsed in distilled water and then stained in aqueous 2% Pb(NO_3_)_3_ (cat. # 228621, Sigma-Aldrich). After rinsing in distilled water and air drying, the sections were subjected to low voltage electron microscopy using the LVEM25 (Delong Instruments, Brno, Czech Republic).

### 2.10. Data Collection and Statistical Analysis

Twenty light microscopic images of cerebral cortex sections of at least 4 animal brains with transient cerebral ischemia were analysed for GHSR1 expression. The intensity of the GHSR1 signals was measured within the cells in arbitrary units (AU) and normalised on the measured cell area. The data sets were subjected to Welch’s *t*-test and are presented as medians ± standard deviation (SD). The sample size of each group is indicated within the corresponding figure legend. For in vitro cultures, images of 200 to 800 cells from each marker per group were taken and subjected to analysis of the intensity signals for each condition. At least four independent cultures obtained from six animals were used per condition. The percentage of cells positive for each marker was calculated and presented as means ± standard error of the mean (SEM). The intensity of the GHSR1 fluorescence signals was measured in AU and normalised on the measured cell area—the data are presented as medians ± SD. All images used for comparison were taken at the same setting on the confocal spinning disc microscope. For dot blot analysis, the chemiluminescent signals of homogenates from organotypic slices of four animals (four independent experimental samples per condition) were processed with the ImageJ software as described above and each GHSR1 signal was referenced to the signal of the corresponding ß-actin band. The data are presented as ratios (medians ± SD). The data sets were subjected to One-Way ANOVA statistical analysis using the post hoc Tukey’s Multiple Comparison Test or Kruskal-Wallis Multiple Comparison Test to assess the statistical significance of differences between the various conditions. The values of *p* < 0.05 were regarded as statistically significant. All *p*-values and sample sizes are indicated in the corresponding figure/figure legends. The statistical analyses were performed with the GraphPad Prism software (version 9.3.0, GraphPad Software Inc., San Diego, CA, USA).

## 3. Results

### 3.1. Experimental Ischemia In Vivo Leads to Increased GHSR1 Expression Levels in Cerebral Cortex Cells

**Figure 1:**
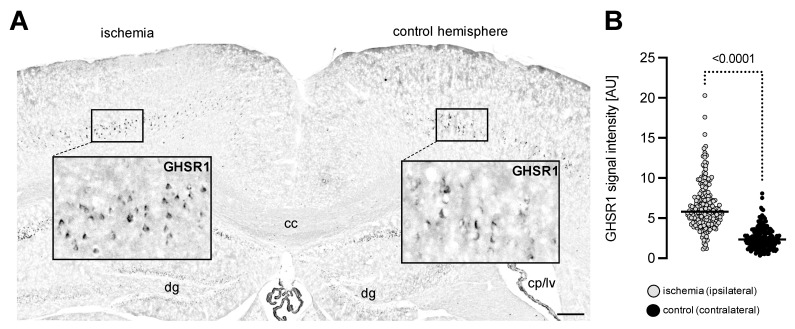
GHSR1 expression in cerebral cortex sections from brains of mice with experimentally induced transient cerebral ischemia. (**A**,**B**) Immunostaining with an antibody recognising GHSR1 (black staining) on a representative coronal brain section showing strong GHSR1-immunoreactivity at the occlusion site (ischemia/ipsilateral) in comparison to the signal of the contralateral side (control hemisphere). Note that the contralateral signal was restricted mainly to the cytoplasm (crescent-shaped appearance). Abbreviations: cc—corpus callosum, dg—dentate gyrus, cp/lv—choroid plexus/lateral ventricle. Scale bar, 300 µm. The GHSR1 signal intensity within the cells was measured in arbitrary units [AU]. Data are presented as medians ± SD analysed by Welch’s *t*-test. The *p*-value is indicated; *n* = 203 measured ipsilateral cells; *n* = 197 measured contralateral cells.

There is a growing body of evidence indicating that ghrelin ameliorates neuroregeneration upon injury. Therefore, we became interested in testing whether transient cerebral ischemia, which was induced experimentally in mice, may alter the GHSR1 expression in cerebral cells within the ischemic region. To this aim, we occluded the middle cerebral artery of adult mice for 45 min and allowed afterwards reperfusion to take place, thus inducing transient cerebral ischemia. Animals were then sacrificed on day 28 after induction of stroke and subjected to immunostaining for the ghrelin receptor GHSR1. Analysis of immunostaining intensity signals revealed enhanced GHSR1 immunoreactivity in cortical cells of the stroke area when compared to the expression observed at the contralateral side (Figure 1A,B). In the ipsilateral hemisphere (occlusion site), the GSHR1 signal was detected across the cerebral cortex cell bodies, whereas contralaterally, the signal was mainly restricted to the cytoplasm (Figure 1A). The quantification analysis indicated that the condition of stroke (hypoxia) stimulated the expression of GHSR1 in cerebral cortex cells.

### 3.2. Experimental Hypoxia In Vitro Leads to Increased GHSR1-Immunoreactivity in Cultured Cerebral Cortex Cells, Cerebellar Granule Neurons and Organotypic Cerebral Cortex Slices

**Figure 2:**
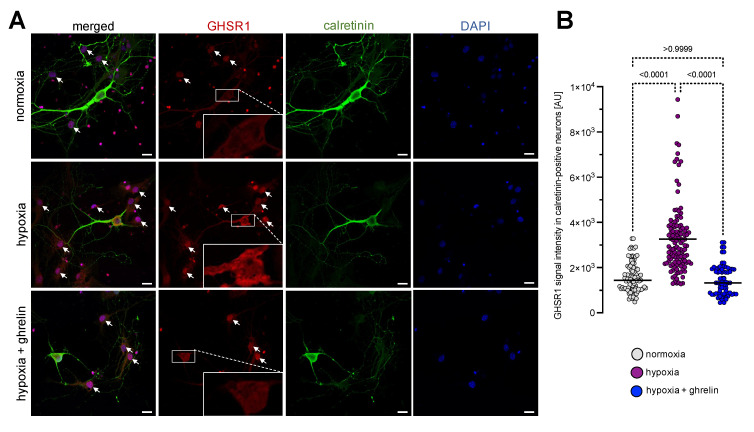
Hypoxia imposed on dissociated cerebral cortex cells stimulated expression of GHSR1. (**A**) Immunostaining with antibodies recognising calretinin (green) and GHSR1 (red) on neonatal dissociated rat cortical neurons that had been cultured for one week and then exposed to severe hypoxia for 6 h, followed by a recovery period of 24 h under normal oxygen supply. DAPI (blue) was used to stain the nuclei. Calretinin immunofluorescence revealed the arborisation pattern of the cerebral cortex neurons, which were surrounded by nonneural and glial cells (arrows). Under normoxic conditions, neurons expressed GHSR1 at the plasma membrane, cytoplasm and nucleus (inlets). Hypoxia imposed on the neurons led to increased expression levels of GHSR1 (**A**,**B**). Note that in the nuclei as well as in the cytoplasm of hypoxic glia and fibroblasts the GHSR1 signal was also increased. Application of ghrelin to hypoxic cultures reduced the expression levels of GHSR1 in neurons to normoxic levels (**A**,**B**). Scale bar, 30 µm. Data are presented as medians ± SD analysed by Kruskal-Wallis’ Multiple Comparison Test. *p*-values are indicated; *n* = 97 for normoxia; *n* = 119 for hypoxia and *n* = 70 for hypoxia + ghrelin. See also Supplementary Figure S1.

Following the results from the experimental in vivo stroke model, we became interested in testing whether the stimulatory effect of hypoxia on the GHRS1 expression could be seen in neuronal cultures deprived of oxygen. As a proof-of-principle, we used an in vitro model of hypoxia consisting of neonatal dissociated rat cortical neurons that had been cultured for one week and then exposed to severe hypoxia for 6 h, followed by a recovery period of 24 h under normal oxygen supply. Half of the cultures were supplemented with ghrelin during these 24 h, while the other half remained untreated as a control. All samples were fixed and processed for immunostaining with antibodies recognising calretinin and GHSR1. We tested the specificity of the GHSR1 antibody in two control staining set-ups: prior to staining of the cell cultures, we incubated the murine primary GHSR1 antibody with murine pituitary/hypothalamus tissue homogenate to opsonise the antibody with detergent-solubilised GHSR1 (Supplementary Figure S1A and Methods). For the second control staining procedure, we applied only the secondary antibody, without the primary GHSR1 antibody (Supplementary Figure S1B and Methods). The signal of calretinin immunofluorescence revealed arborised neurons, which were surrounded by nonneural (fibroblasts) and glial cells (Figure 2A, arrows). The GHSR1 fluorescence signal in calretinin-positive neurons was measured (Figure 2B). Under normoxic conditions, the neuronal immunoreactivity for GHSR1 was weak at the plasma membrane, cytoplasm and nucleus (Figure 2A). Hypoxia imposed on the cell cultures led to increased GHSR1-immunoreactivity in calretinin-positive neurons (Figure 2A,B). The GHSR1 signal was increased in the neurites and nuclei of the neurons. Of note, in the nuclei as well as in the cytoplasm of hypoxic glia and fibroblasts the GHSR1 signal was also increased (Figure 2A, arrows, middle panel). When ghrelin was applied to hypoxic neurons, the fluorescence signals of GHSR1 in neurons were reduced to those measured under normoxia (Figure 2A,B).

**Figure 3:**
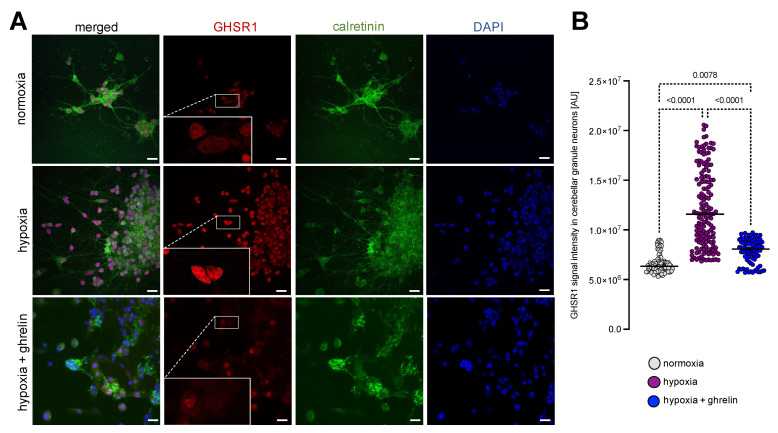
Hypoxia imposed on dissociated cerebellar granule neurons stimulated nuclear expression of GHSR1. (**A**) Immunostaining for calretinin (green) and GHSR1 (red) on cerebellar granule neurons that had been cultured for 24 h and then exposed to severe hypoxia for 6 h, followed by a recovery period of 24 h under normal oxygen supply. DAPI (blue) was used to stain the nuclei. Cerebellar granule neurons were unipolar, partially growing in clusters. In normoxic cerebellar granule neurons, the expression of GHSR1 was predominantly restricted to the nucleus (inlets). Hypoxia led to increased GHSR1-immunoreactivity in the nucleus (**A**,**B**). When ghrelin was applied to recovering hypoxic cerebellar granule neurons, the GHSR1 signals were similar to the normoxic levels (**A**,**B**). Scale bar, 30 µm. Data are presented as medians ± SD analysed by Kruskal-Wallis’ Multiple Comparison Test. *p*-values are indicated; *n* = 99 for normoxia; *n* = 151 for hypoxia and *n* = 83 for hypoxia + ghrelin.

We next asked whether these effects hold true for other types of neurons and studied, therefore, dissociated rat cerebellar granule neurons that had been cultured for 24 h and then exposed to severe hypoxia for 6 h, followed by a recovery period of 24 h under normal oxygen supply. Similar to the experimental design with cortex neurons mentioned above, half of the cultures of cerebellar granule neurons were supplemented with ghrelin during the recovery phase, while the other half remained untreated as a control. All samples were fixed and immunostained for calretinin and GHSR1 (Figure 3A). The calretinin immunostained cerebellar granule neurons appeared unipolar, often growing in clusters (Figure 3A). In normoxic cerebellar granule neurons, the expression of GHSR1 was weak and predominantly restricted to the nucleus (Figure 3A, inlets). Hypoxia led to increased GHSR1-immunoreactivity in the nucleus (Figure 3A,B). When ghrelin was applied to recovering hypoxic cerebellar granule neurons, the GHSR1 signals were reduced to those measured under normoxia (Figure 3A,B).

**Figure 4:**
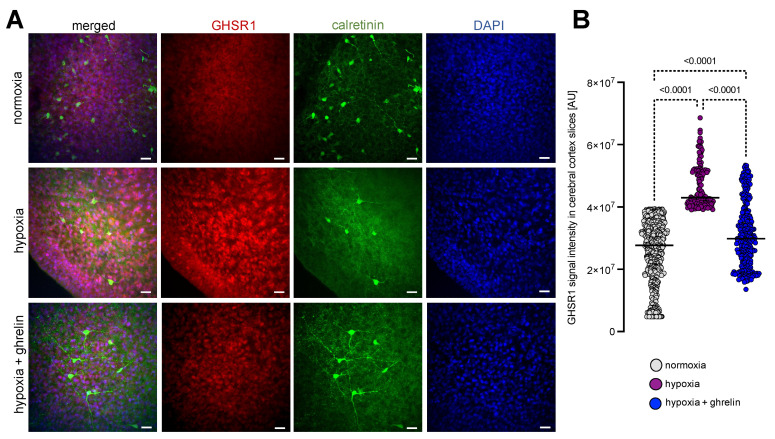
Hypoxia on acute cerebral cortex slices stimulated expression of GHSR1. (**A**) Calretinin (green) and GHSR1 (red) immunostaining of cerebral cortex slices. Slices were obtained from 4-day-old rats and cultured for 24 h to be then exposed to severe hypoxia for 6 h, followed by a recovery period of 24 h under normal oxygen supply. DAPI (blue) was used to stain the nuclei. In slices, calretinin-positive cerebral cortex neurons appeared multipolar, surrounded by many calretinin-negative cells expressing GHSR1. Normoxic expression of GHSR1 was homogeneously distributed across the sliced tissue. Hypoxia imposed on the slices led to increased GHSR1-immunoreactivity (**A**,**B**). When ghrelin was applied to recovering hypoxic slices, the GHSR1 signals were slightly reduced (in neurons almost to normoxic levels). Many calretinin-negative cells were still strongly positive for GHSR1. Scale bar, 30 µm. Data are presented as medians ± SD analysed by Kruskal-Wallis’ Multiple Comparison Test; *p*-values are indicated; *n* = 562 for normoxia; *n* = 142 for hypoxia and *n* = 274 for hypoxia + ghrelin.

In parallel, we prepared acute organotypic slice cultures from brain hemispheres of 4-day-old Wistar rats and exposed the slices to severe hypoxia for 6 h, followed by a recovery period of 24 h under normal oxygen supply. One half of the cerebral cortex slices were supplemented with ghrelin during the recovery phase, while the other half remained untreated as a control. After fixation, the samples were immunostained for calretinin and GHSR1 (Figure 4A). In slices, the calretinin immunostained cerebral cortex neurons appeared multipolar, surrounded by a huge population of calretinin-negative cells expressing GHSR1 (Figure 4A). Under normoxia, the GHSR1 signal was homogeneously distributed across the sliced tissue (Figure 4A). Hypoxia imposed on the slices led to increased GHSR1-immunoreactivity (Figure 4A,B). When ghrelin was applied to recovering hypoxic slices, the GHSR1 signals were slightly reduced (in neurons almost to those seen under normoxia) (Figure 4A,B). However, many calretinin-negative cells were still displaying strong GHSR1 signals.

**Figure 5:**
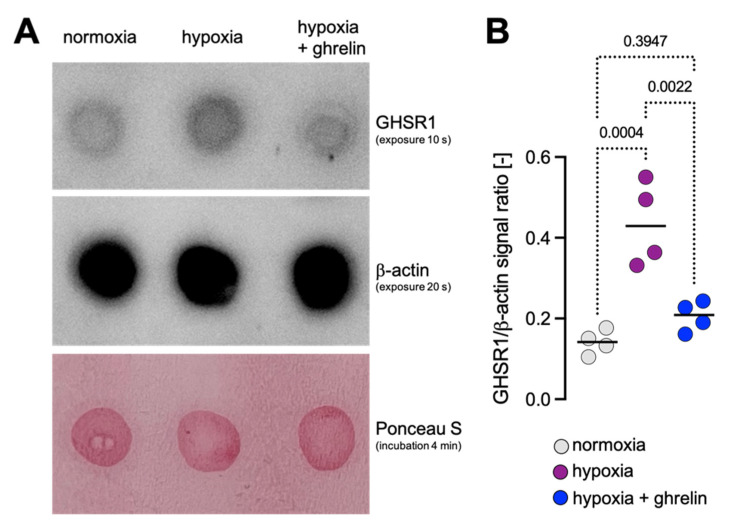
Dot blot analysis of homogenates from cerebral cortex slices under normoxic and hypoxic conditions. (**A**) Dot blots with homogenates from slices under normoxia, hypoxia and hypoxia followed by ghrelin treatment. Antibodies recognising GHSR1 and β -actin were used. Lower panel: Ponceau S staining. Exposure/incubation times are indicated. (**A**,**B**) Quantitative analysis of the dots confirmed that the expression of GHSR1 was increased in the homogenates of the slices which were subjected to hypoxia in comparison to normoxic slices. Ghrelin application to recovering hypoxic slices decreased the levels of GHSR1. Data are presented as medians ± SD analysed by One-Way ANOVA with Tukey’s Multiple Comparison Test; *p*-values are indicated; *n* = 4 for normoxia; *n* = 4 for hypoxia and *n* = 4 for hypoxia + ghrelin. See also Supplementary Figure S2.

Furthermore, we prepared homogenates from the slices and subjected the homogenates to dot blot analysis using the GHSR1 antibody. Protein loading was controlled by immunodetection for β -actin and by staining with Ponceau S (Figure 5A and Figure S2). The GHSR1 signal was normalised on the β -actin signal. The quantitative analysis of the dots confirmed that the expression of GHSR1 was increased in the homogenates of the slices which were subjected to hypoxia when compared to normoxic slices (Figure 5A,B and Figure S2). Ghrelin application to slices recovering from hypoxia decreased the levels of GHSR1 (Figure 5A,B and Figure S2).

**Figure 6:**
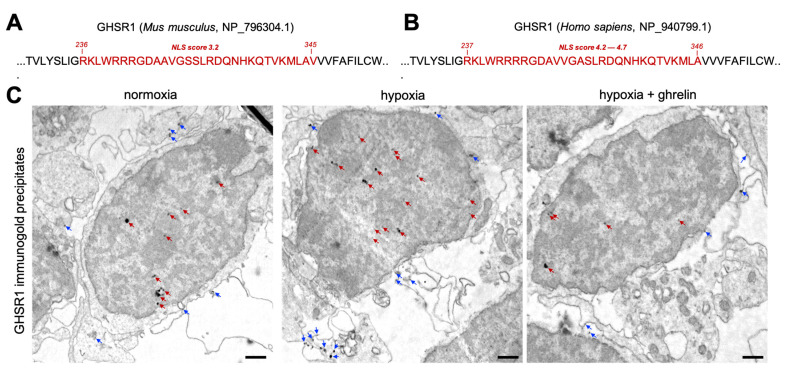
Nuclear presence and chromatin association of GHSR1. (**A**,**B**) Amino acid sequences of the murine and human GHSR1 with predicted nuclear localisation signal regions (NLS, in red). Initial and end positions of the NLS are indicated, the NLS prediction scores are given. (**C**) Transmission electron microphotographs showing the intracellular distribution of GHSR1-immunogold precipitates/grains in normoxic, hypoxic and ghrelin-treated hypoxic cerebral cortex cells. Blue arrows indicate immunogold precipitates found within the cytoplasm, on vesicles and on the plasma membrane. Red arrows indicate nuclear GHSR1-immunogold precipitates (predominantly associated with the heterochromatin). Note the increased number of cytoplasmic and nuclear gold grains under the condition of hypoxia. Scale bar, 100 nm.

Considering the GHSR1 immunoreactivity found on the cell surface and within the cell interior, we asked whether the receptor is present in the cell nuclei and associates with the chromatin. We first mapped the murine and human GHSR1 protein sequence for nuclear localisation signals in the cNLS Mapper [28]. The predicted NLS in the mouse GHSR1 sequence (Figure 6A) and that of the human (Figure 6B) received the scores 3.2 and 4.2–4.7, respectively. Such scores suggest a potential cytoplasmatic and nuclear presence of GHSR1 [28]. Following these predictions, we performed immunogold transmission electron microscopy for GHSR1 on cerebral cortex cells under normoxia, hypoxia and hypoxia with ghrelin treatment (Figure 6C). Under normoxic conditions, we found a few immunogold grains/precipitates within the cytoplasm and the nucleus of the cells (Figure 6C, first panel), whereas, under hypoxia, the amount of the immunogold precipitates was per se higher in both the cytoplasm and nucleus (Figure 6C, second panel). In the hypoxic cells that had received ghrelin, the amount of cytoplasmic and nuclear immunogold precipitates appeared reduced (Figure 6C, third panel). Of note, in all three conditions, nuclear immunogold precipitates were found in the regions of the heterochromatin (Figure 6C, red arrows). Interestingly, under hypoxia, a few gold grains were found also within the euchromatin. These combined findings confirmed the nuclear presence and the chromatin association of GHSR1.

### 3.3. Experimental Hypoxia In Vitro Leads to Decreased Numbers of Pax6 and NeuN Expressing Cerebral Cortex Cells, but Increases the Number of Ki67-Positive Cells

**Figure 7:**
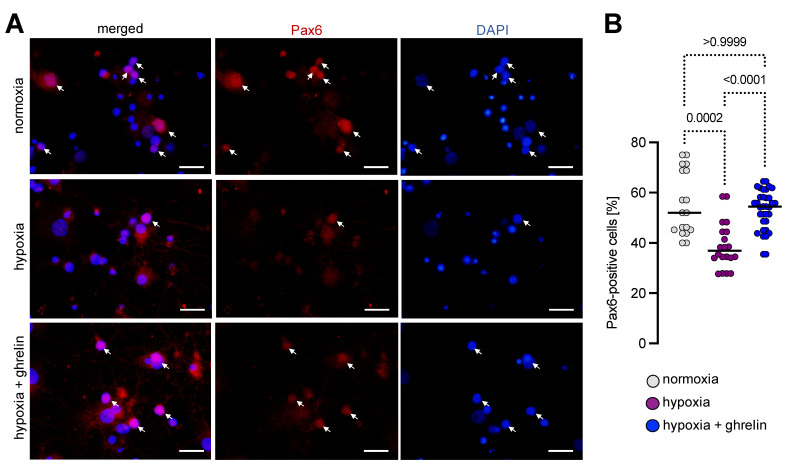
Hypoxia applied to dissociated cerebral cortex cells reduced the number of Pax6 expressing cells. (**A**,**B**) Pax6 immunostaining of dissociated cerebral cortex cells (in a mature state) under normoxic and hypoxic conditions. Approximately half of the matured cortical cultures were Pax6-positive (arrows), whereas hypoxia significantly decreased their proportion. Ghrelin treatment after exposure to hypoxia upregulated the number of Pax6 expressing cells to normoxic levels. Scale bar, 20 µm. Data are presented as medians ± SD analysed by Kruskal-Wallis’ Multiple Comparison Test; *p*-values are indicated; *n* = 18 for normoxia; *n* = 20 for hypoxia and *n* = 34 for hypoxia + ghrelin.

Based on the findings obtained from the transmission electron microscopy, we hypothesised that nuclear GHSR1 affected the expression of neurogenic and mitotic factors such as Pax6 and Ki67. To test this hypothesis, we prepared neonatal dissociated rat cerebral cortex neurons that had been cultured for 3 weeks to a mature stage to then expose them to severe hypoxia for 6 h, followed by a recovery period of 24 h under normal oxygen supply. Half of the cultures were supplemented with ghrelin during 24 h of recovery, while the other half remained untreated as a control. We immunostained the cultures for Pax6 (Figure 7A). Because of the relative difference in the cell density, we calculated the Pax6-positive cells as a percentage of all cells (counterstained with DAPI). In mature 3-week-old cultures under normoxic conditions, 55.5 ± 3.0% (mean ± SEM) of the cells expressed Pax6 (Figure 7A,B). Their proportion was decreased under hypoxia to 38.9 ± 2.1% (mean ± SEM), whereas ghrelin treatment upregulated the expression levels of Pax6 (53.2 ± 1.4%, mean ± SEM) and brought them closely to levels seen under normoxia (Figure 6B).

**Figure 8:**
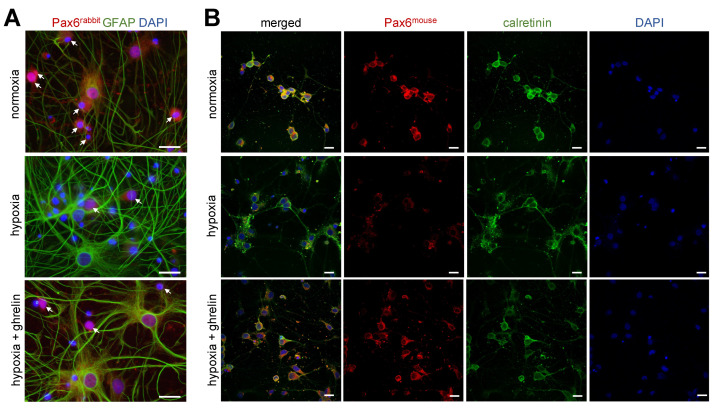
Hypoxia imposed on 3-week-old cerebral cortex cells led to decreased immunoreactivity for Pax6. (**A**) Pax6 (rabbit) and GFAP immunostaining of dissociated cerebral cortex cells (in a mature state) under normoxic and hypoxic conditions. Normoxic GFAP-positive cells (glial cells) were also positive for Pax6 and surrounded by many GFAP-negative cells with Pax6-immunoreactivity (arrows). Hypoxia lowered the number of GFAP/Pax6-double-positive cells, whereas, ghrelin application after hypoxia increased this number to normoxic levels (means ± SEM for normoxia: 25.6 ± 3.9%, *n* = 11; hypoxia: 14.7 ± 2.1%, *n* = 10; hypoxia + ghrelin: 30.9 ± 6.0%, *n* = 11; *p* < 0.0001, One-Way ANOVA with Tukey’s Multiple Comparison Test). Note the overall weak Pax6-immunoreactivity signals in hypoxic neurons vs. the condition of normoxia and hypoxia + ghrelin. Scale bar, 20 µm. (**B**) Pax6 (mouse) and calretinin immunostaining of matured dissociated cerebral cortex cells revealed weak Pax6-immunoreactivity in hypoxic calretinin-positive neurons when compared to normoxic and ghrelin-treated hypoxic neurons. (**A**,**B**) DAPI was used to stain the nuclei. Analysis of the nuclear Pax6 signal intensity (medians ± SD in arbitrary units) for normoxia: 1.62 ± 0.69, *n* = 50; hypoxia: 0.77 ± 0.59, *n* = 52; hypoxia + ghrelin: 1.15 ± 0.58, *n* = 52; *p* < 0.0001, Kruskal-Wallis’ Multiple Comparison Test. Scale bar, 30 µm.

Additionally, staining of 3-week-old cerebral cortex cell cultures for Pax6 (produced in rabbit) and GFAP was performed (Figure 8A). Under normoxic conditions, 25.6 ± 3.9% (mean ± SEM) of the cultured cells expressed both Pax6 and GFAP (Figure 8A, upper panel). In the hypoxic group 14.7 ± 2.1% (mean ± SEM) of the cells were positive for Pax6 and GFAP (Figure 8A, middle panel). In hypoxic cultures supplemented with ghrelin during the recovery period, 30.9 ± 6.0% (mean ± SEM) of all cells were double-stained for Pax6 and GFAP (Figure 8A, lower panel). In a parallel set-up of the same conditions, we used another Pax6 antibody (produced in the mouse) in combination with calretinin (Figure 8B). Normoxic neurons stained for calbindin were strongly immunoreactive for Pax6 (mouse), whereas hypoxic neurons displayed weak immunoreactivity signals (Figure 8B, upper and middle panels). Ghrelin-treated hypoxic neurons showed strong Pax6-immunoreactivity signals (Figure 8B, lower panels).

**Figure 9:**
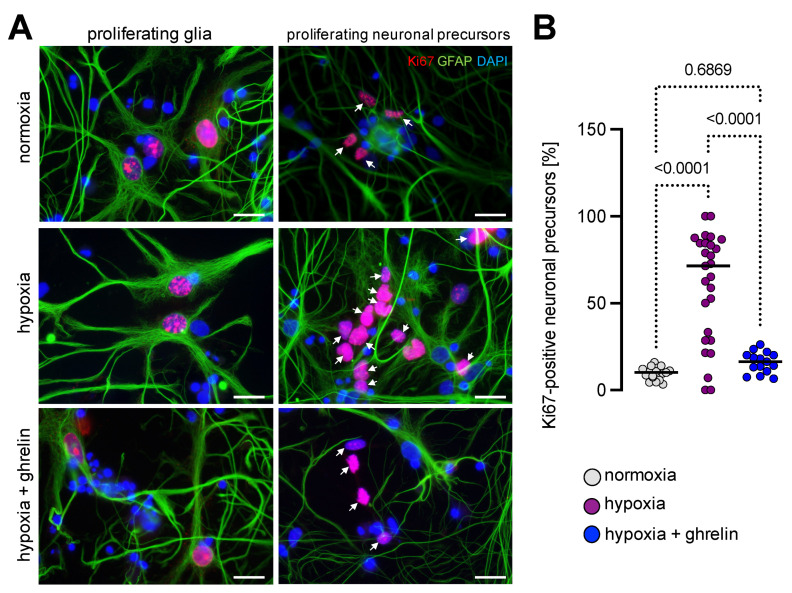
Hypoxia imposed on dissociated cerebral cortex neurons stimulated the proliferation of neural precursors. (**A**) Immunolabelling of dissociated cortical neurons for Ki67 (pink after merging with DAPI) and GFAP (green) revealed the complex network of glial cells and neurons. DAPI was used to stain the nuclei. Proliferating glial cells were positive for Ki67 and showed a speckled pattern of nuclear staining for the antigen, surrounded by GFAP-stained fibrils (left panels). The double staining showed also that the majority of Ki67-positive cells under hypoxic conditions were neuronal precursors (right panels, arrows). Scale bar, 20 µm. (**B**) Quantitative analysis revealed that hypoxia significantly elevated the number of Ki67-positive neuronal precursors, whereas ghrelin treatment reduced these numbers to normoxic values. Data are presented as medians ± SD analysed by Kruskal-Wallis’ Multiple Comparison Test. *p*-values are indicated; *n* = 18 for normoxia; *n* = 27 for hypoxia and *n* = 15 for hypoxia + ghrelin.

We further immunostained the cultures for Ki67 (Figure 9A,B) to detect active cell proliferation. The pattern of staining varied significantly between the cells; in some of them, the staining signal was restricted to the nucleoli, whereas in the large nuclei the signal appeared dispersed throughout the entire nucleus (Figure 9A,B). Moreover, some of the nuclei showed speckled patterns of staining (e.g., glial cells, Figure 9A). Proliferating neuronal precursors displayed Ki67-positive nuclei smaller than those of the proliferating glial cells, localised in the close proximity of the GFAP-positive glial cell nets. Under normal oxygen supply, the Ki67 index (percentage of neuronal precursors positive for the Ki67 antigen) was 9.7 ± 0.8% (mean ± SEM). Interestingly, in control cultures exposed to hypoxia for 6 h and followed by 24 h of re-oxygenation, the proportion of proliferating neuronal precursors was 59.8 ± 1.4% (mean ± SEM). In the experimental group supplemented with ghrelin during the recovery period, Ki67 was detected in 15.9 ± 1.6% (mean ± SEM) of the neuronal precursor nuclei (Figure 9A,B).

**Figure 10:**
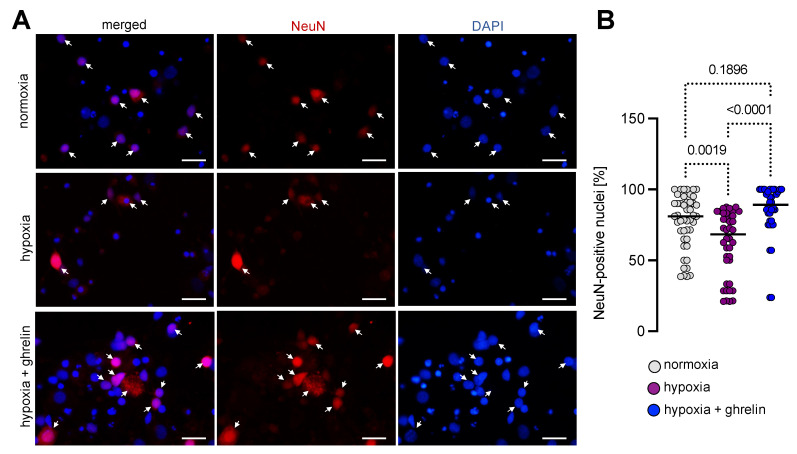
Hypoxia decreased the number of NeuN-expressing post-mitotic neurons. (**A**) NeuN immunolabelling of post-mitotic dissociated cortical neurons (arrows). DAPI was used to stain the nuclei. NeuN was expressed not only in the nuclei of the neurons but also in the cytoplasm of many of them. Additionally, some neurons showed a higher level of antigen expression than others. Scale bar, 20 µm. (**B**) Hypoxia decreased the ratio of NeuN-positive neurons, whereas post-hypoxic application of ghrelin increased the number of NeuN-positive neurons to normoxic values. Data are presented as medians ± SD analysed by Kruskal-Wallis’ Multiple Comparison Test. *p*-values are indicated; *n* = 46 for normoxia; *n* = 40 for hypoxia and *n* = 36 for hypoxia + ghrelin.

Further immunostaining for visualisation of the marker for post-mitotic neurons, NeuN, revealed immunoreactivity not only in the nuclei but also in the cytoplasm of many neurons, including the initial part of the neurites (Figure 10A). Some cells exhibited a strong immunoreactivity for the antigen (Figure 10A). The comparison between the quantitative data pointed to a significant decrease in the ratio of the NeuN-positive neurons under hypoxia (Figure 10B). Post-hypoxic supplementation of ghrelin increased the number of NeuN-positive neurons to normoxic values (Figure 10A,B). In particular, we observed that hypoxia reduced significantly the ratio of NeuN-positive post-mitotic neurons (61.5 ± 3.7%, mean ± SEM) compared with the ratio which was assessed under normoxia (77.1 ± 2.8%, mean ± SEM). Ghrelin treatment during the post-hypoxic period elevated the fraction of neurons positive for NeuN up to numbers observed under normoxia (85.1 ± 3.2%, mean ± SEM).

## 4. Discussion

The main findings of the present study are: (i) The expression of ghrelin’s receptor GHSR1 is enhanced after hypoxia-induced in vivo and In vitro; (ii) Ghrelin treatment of hypoxic cultures counteracts the increase of GHSR1; (iii) hypoxia reduces the Pax6 levels In vitro, simultaneously increasing neural progenitor proliferation.

In some areas of the nervous system, such as the hypothalamus and the spinal cord, ghrelin has been shown to play an important role in (adult) neurogenesis [29,30]. Interestingly, ghrelin is the only known endogenous ligand activating the GHSR1 [31,32]. In contrast to ghrelin, GHSR1 is highly expressed in the nervous system [33,34,35,36,37,38]. Does the expression level of GHSR1 change upon injury or stress imposed on the nervous system? Cabral et al. [39] have suggested that neuronal remodelling is GHSR1-mediated and depends on the energy balance. Moreover, experimental evidence from the past two decades has revealed that ghrelin mediates neuroregeneration upon injury (for a detailed review see [40]). Therefore, we became interested in testing whether neurons subjected to metabolic stress may alter their GHSR1 expression. We demonstrated that hypoxia imposed on the cell cultures led to increased GHSR1-immunoreactivity in the neurites and nuclei of the neurons, as well as in the cytoplasm and nuclei of hypoxic glia and fibroblasts. On the contrary, previous studies have reported downregulation of GHSR1 expression after ischemia [18,41], and controversial effects of ghrelin on GHSR1′s expression—while the research group of Miao [18] reported that postischemic treatment of rats with ghrelin led to an elevation of the receptor levels, Huang et al. [41] did not observe any change. These discrepancies could be due to the methods of sampling or/and the way of exposure of the cultures or model systems to ischemia/hypoxia, or/and the concentration of ghrelin utilised for administration.

The methods of hypoxia inducement and ghrelin application in vivo reported in the literature vary significantly and lead to controversial experimental outcomes. The concentration of 0.5 µM used in our experiments complies with that reported by us [27,42] and other groups [24,26,43,44,45], yet differs from the concentrations utilised by Miao et al. [18] and Huang et al. [41]. Nevertheless, we abode by our previously reported concentrations because of the probability that the acute slices might require more ghrelin to recover after hypoxia: i.e., being placed on membranes, they are nourished through diffusion from the medium underneath, where ghrelin was applied. For the sake of consistency, we used 0.5 µM of ghrelin in all experiments. 

Ghrelin has been shown to exert neuroprotection via attenuation of oxidative stress [41], blockade of apoptosis [18], and stimulation of synaptic plasticity [46]. Suda et al. [47] have suggested that defective GHSR1 activity of dopaminergic neurons causes marked motor impairment. Remarkably, mice deficient in GHSR1 did not display any signs of neurodegeneration [48]. We interpret the upregulation of GHSR1 upon hypoxia to be a response to hypoxia/injury/stress. 

The plasma levels of ghrelin are relatively low [49], therefore, the reaction of neurons upon hypoxia with increased levels of GHSR1 could have an amplifier-like effect for low concentrations of ghrelin (see also [40]). To the best of our knowledge, the finding that hypoxia upregulates the expression levels of GHSR1 in neurons is novel. So is also the finding that GHSR1 is present in/translocates to the nucleus and associates with the chromatin. The chromatin association of GHSR1 might affect the expression of transcription factors.

Pax6 is a crucial factor determining the fate of neuronal progenitors in vitro [14] as well as in vivo, in adult brains [50]. However, the capacity of Pax6 to efficiently reprogram nonneuronal cells into neurons, observed in vitro, is limited in situ in the adult brain due to the natural postnatal glycogenic environment [51,52,53]. Under pathological conditions, such as hypoxia, Pax6 expression is upregulated in the neurogenic niches but not in the cortex, as shown in rats enduring prenatal hypoxia [2]. Thus, the reduced expression of Pax6 in dissociated mature cortical cells in vitro under hypoxic conditions (38.9 ± 2.1%) observed in our study is in line with the published phenomenon in vivo. On the other hand, post-hypoxic exposure to ghrelin for 24 h significantly elevated the percentage of cells expressing Pax6 (53.2 ± 1.4%), bringing it to the level at the pre-hypoxic stage (55.5 ± 3.0%). To our knowledge, this is the first study demonstrating such a modulatory effect of ghrelin on Pax6 expression in mature cortical networks. Of note, Fong et al. [54] have shown that proliferation and maturation of neuroblast cell lines can be stimulated via neurotrophins and neuroprotective factors derived from cocultured C17.2 neural precursor cells. In our study, the marker of proliferating precursor cells Pax6 was found in GFAP and calretinin-immunoreactive cells. The number of Pax6-positive cells significantly exceeded the number of Pax6/GFAP-double-labelled cells, which indirectly indicates that not only astrocytes express the transcription factor but other cell types are positive as well. Indeed, double labelling for Pax6 and calretinin confirmed that neurons in mature cortical cultures express Pax6. These results are in consent with the findings of Nacher et al. [55] that Pax6-expressing cells are also present in the adult hippocampal dentate gyrus and in the subventricular zone/rostral migratory stream, and they are proliferating precursors as well as nonproliferating resting progenitor cells or granule neurons at a very early developmental stage. Strikingly, hypoxia reduced the number of Pax6-positive cells (glial cells and neurons/neuronal precursors), whereas ghrelin treatment restored their levels. These observations raised two questions, which need further attention: does ghrelin act as a trophic factor; is the expression of Pax6 temporally and spatially regulated by ghrelin? 

During development, for instance, Pax6 expression is graded in time and space: the highest level of expression in the cortical neuroepithelial cells is at the onset of neurogenesis and lack of it in the basal progenitors of the subventricular zone and in the post-mitotic neurons [56]. The same grading also refers to the Pax6-mediated control of cell cycle’s duration in cortical progenitors. Primarily, Pax6 has a repressive effect on the cell cycle progression [5]. Lack of Pax6 at early stages of mice development (embryonic day 12.5) results in shortening of the progenitor cell cycle and a temporary increased production of post-mitotic neurons [57,58], whereas at later stages (embryonic day 15.5), Pax6-deficient progenitors proliferate more slowly [59]. Thus, even in the same cell type, Pax6 can obviously do both: the factor promotes and inhibits the proliferation of cerebral progenitors [17,58,60]. In the adult pancreas, Pax6-deficient beta- and alpha-cells have been shown to lose their maturation characteristics and convert into ghrelin positive cells [61]. These observations suggest that the effects of Pax6 on the cell cycle (e.g., of the cortical progenitors) are context-dependent as supported by the concept that the transcription factor affects proliferation in opposite manners via its DNA-binding subdomains [62].

The Pax6 protein comprises two distinct DNA-binding domains, one of which is regulated by alternate splicing and is able to interact with a number of co-factors [5]. Recently, Brg1/Brm associated factors complex (BAF) and Meis2 have been identified as new co-factors modulating Pax6′s direct regulation of target gene expression during embryonic and adult neurogenesis [63,64,65]. Importantly, Pax6 is also expressed in glial progenitors in the adult brain [66,67]. We speculate that ghrelin functions as a co-factor modulating Pax6 expression, possibly affecting neural progenitor cell fate or cell cycle. However, to explore whether this modulatory effect of ghrelin is mediated directly via the transcription factor Pax6, or whether there is another yet unknown mechanism, additional experiments are needed.

The Ki67 antigen is expressed in the nuclei exclusively in proliferating cells [68,69]. Quiescent or resting cells in the G_0_ phase of the cell division cycle do not express it, therefore Ki67 is considered as a marker for the so-called growth fraction of given cells, i.e., progenitor cells [70,71]. During the interphase, cells express Ki67 exclusively in the nucleus, where it is required for the normal distribution and nucleolar association of the heterochromatin [72]. During mitosis, Ki67 covers the surface of chromosomes and represents approximately one-third of their protein mass [73]. Ki67 prevents chromosomal aggregation [74] and asymmetric distribution in daughter cells [75]. The transcription factor increases during the S phase, with further escalation in the G_2_ phase, and maximal intensity in the metaphase [76,77]. The half-life of Ki67 is estimated to be approximately 1 h [68]. Intriguingly, cells entering the G_0_ phase when treated with growth factors can re-increase their Ki67, to re-enter the S phase [76]. During the early G_1_ phase, Ki67 is multi-focally expressed throughout the nucleoplasm, while during the S and the G_2_ phases, larger foci overlapping the nucleoli and the heterochromatin regions are formed [77]. After the disintegration of the nuclear envelope, part of the Ki67 protein could be observed dispersed in the cytoplasm [77]. These patterns, including the chromosomes covered by the Ki67 protein, were also observed in our experiments. Before the onset of hypoxia, we found that 9.7 ± 0.8% of the cultured neuronal progenitor cells expressed Ki67, and their proportion was surprisingly increased up to 59.8 ± 1.4% after hypoxia. Ghrelin administration downregulated the percentage of Ki67-positive neural progenitors to 15.9 ± 1.6%. Our findings contradict the results from other studies showing that ghrelin administration in animal models of chronic neurodegenerative diseases, as well as endogenous ghrelin in other in vivo and in vitro experiments, triggers adult neurogenesis: ghrelin has been shown to stimulate proliferation of progenitor cells and increase the number of immature neurons in the hippocampus and the subventricular zone [78,79,80]. However, to the best of our knowledge, the current proof-of-principle study is the first one demonstrating that ghrelin attenuates the proliferation of progenitor cells in the cerebral cortex. Nevertheless, it is important to mention in this respect that Belaev and collaborators [81] have observed continuous proliferation of newly born neural precursors after occlusion of the middle cerebral artery of rats for 2 h. Treatment of the animals with docosahexaenoic acid (DHA) (member of the omega-3 polyunsaturated fatty acids family) 1 hour after the occlusion, followed by immunostaining two weeks post-injury, showed a neuroprotective and neurogenic effect, resulting in 88% increase of the BrdU/Ki67-positive and BrdU/NeuN-positive cortical cells in the penumbra. On the other hand, it seems that the Ki67 protein expression also depends on the degree of hypoxia, as established in experiments with glioma-derived neurospheres. Hypoxia limited to oxygen levels lower than 2% induces high proliferation rates [82], yet if the values are lowered to less than 1% oxygen, a decrease of the Ki67 antigen expression occurs [83].

Additionally, we have to carefully extrapolate the results from rodent models to non-human- and human primates due to the potential interspecies differences in adult neurogenesis. Studies on brains of adult macaque monkeys subjected to global cerebral ischemia have indicated that in both the striatum and neocortex putative newly generated neurons with long-term survival are slightly above 1% [84,85]. In adult human brains, hypoxia triggers some proliferation underneath the ependymal layer, but more than 30% of the Ki67 immunoreactivity is expressed in astrocytes, as double labelling for GFAP and Ki67 has indicated [86]. These GFAP-expressing neural stem cells can differentiate into astrocytes, oligodendrocytes and neurons in the presence [87,88] or absence of exogenous mitogens [89], thus contributing locally to the generation of new neurons as a reaction to ischemic injury. The rapid changes in the Ki67 protein expression, when comparing the normoxic neurons with those of the post-hypoxic experimental condition, can explain the significant fluctuations in the percentage of Ki67-positive cells in a relatively short time frame (24 h). Our findings are consistent with those obtained from human brains studies, where a strong upregulation of Ki67 expression has been observed in the subventricular zone of patients who died just a few days after ischemic stroke, but not in those who died six months after ischemic injury [86]. We interpret the increase in cell proliferation after ischemic injury as a transient event. Of note, Pax6 can be expressed in both neuronal and glial progenitors, and thus, the postischemic reduction of Pax6-positive cells observed in our study might include both cell types.

As Ortega et al. have reported [90], BrdU incorporation did not change after 6 h of hypoxia upon human cortical radial glial cells, but 24 h reduction of oxygen supply limited it, because hypoxia beyond 20 h of duration inflicts irreversible changes within the cells [91]. It appears that the observed effects of hypoxia vary in dependence on the experimental parameters such as location (i.e., cerebral cortex vs. subventricular zone), the severity of hypoxia, and duration of recovery. 

Notably, the results from studies on NeuN in stroke models are quite controversial. Some of them report a substantial decrease of NeuN-positive cells in the infarction core and relate it to neuronal death [92], while others have related this decrease to depletion of NeuN’s expression or loss of antigenicity [93]. In the present study, there was a significant change in the NeuN expression when comparing the three experimental groups, i.e., normoxia (77.1 ± 2.8%), hypoxia (61.5 ± 3.7%) and hypoxia + ghrelin (85.1 ± 3.2%). Of note, the nuclear NeuN staining was substantially reduced or even undetectable in some neurons. Mild cerebral ischemia has the same effect in in vivo experiments; however, it seems that the NeuN protein levels do not change within 24 h post-reperfusion, merely the NeuN antigenicity is reduced. Of note, the proportion of NeuN-positive neurons in the penumbra have been found to partially restore after antigen retrieval [93]. It is also possible that some special cell types or neurons with a distinct physiological/pathological status can be distinguished through the differences in NeuN/Rbfox3 expression [94]. In our experiments, ghrelin supplementation to recovering hypoxic cultures increased the proportion of neurons with nuclear expression of NeuN to pre-hypoxic values. Only more severe injuries, such as axotomy, have been shown to significantly abolish the NeuN protein levels, which then begin to restore within 7 days post-injury to reach the uninjured levels after 28 days [95]. Injured, but still viable neurons, may lose NeuN-protein expression due to downregulated protein synthesis and/or protein overconsumption [96]. Lower production of NeuN/Rbfox3 and the translocation of NeuN/Rbfox3 from the nucleus to the cytoplasm might lead to downregulation of the alternative splicing of the RNA of its target genes, and thus, change the complement of neuronal specific gene expression [97]. Differential splicing is an important mechanism for the evolutionary dynamics of the central nervous system’s complexity [98] and its regular development. NeuN/Rbfox3-regulated splicing is crucial for the final neuronal differentiation during development [99], but mutations in NeuN/Rbfox3 are also associated with neuropsychiatric disorders [100]. Thus, the up-regulating effect of ghrelin on the nuclear expression of NeuN observed in this study suggests that ghrelin can influence neuronal differentiation operating on its master switch [101].

## 5. Conclusions

In conclusion, our in vitro findings suggest that ghrelin is involved in postischemic neuroplasticity in non-neurogenic brain areas such as the cerebral cortex. Expression of the ghrelin receptor GHSR1 is increased in hypoxic neurons and normalised whenever ghrelin is supplemented to these neurons. Moreover, ghrelin regulates Pax6 and Ki67 expression, and stimulates cytoplasmic/nuclear shuttling of NeuN in hypoxic neurons. Since these factors are key players in proliferation, neurogenesis and differentiation, their stimulation could ameliorate recovery after hypoxia. This work emphasises the relevance of additional studies on the regulatory role of ghrelin in the cell cycle of cortical progenitors and neurons exposed to hypoxia, and corroborates the therapeutic potential of ghrelin for the treatment of stroke patients.

## Data Availability

The datasets in support of the findings of this study are available from the corresponding authors, upon reasonable request.

## Supplementary Materials

The following supporting information can be downloaded at: https://www.mdpi.com/2073-4409/11/5/782/s1, Figure S1: Control immunostaining experiments for the GHSR1 antibody referring to Figure 2 of the main text; Figure S2: Uncropped dot blot images referring to Figure 5 of the main text. Areas shown in the main figures and used for quantification are indicated.

## References

[1] Lipton P. (1999). Ischemic cell death in brain neurons. Physiol. Rev..

[2] So K., Chung Y., Yu S.K., Jun Y. (2017). Regional Immunoreactivity of Pax6 in the Neurogenic Zone After Chronic Prenatal Hypoxia. Vivo.

[3] Daval J.L., Vert P. (2004). Apoptosis and neurogenesis after transient hypoxia in the developing rat brain. Semin. Perinatol..

[4] Arvidsson A., Collin T., Kirik D., Kokaia Z., Lindvall O. (2002). Neuronal replacement from endogenous precursors in the adult brain after stroke. Nat. Med..

[5] Manuel M.N., Mi D., Mason J.O., Price D.J. (2015). Regulation of cerebral cortical neurogenesis by the Pax6 transcription factor. Front. Cell. Neurosci..

[6] Seri B., Herrera D.G., Gritti A., Ferron S., Collado L., Vescovi A., Garcia-Verdugo J.M., Alvarez-Buylla A. (2006). Composition and organization of the SCZ: A large germinal layer containing neural stem cells in the adult mammalian brain. Cereb. Cortex.

[7] Van Kampen J.M., Hagg T., Robertson H.A. (2004). Induction of neurogenesis in the adult rat subventricular zone and neostriatum following dopamine D3 receptor stimulation. Eur. J. Neurosci..

[8] Bernier P.J., Bedard A., Vinet J., Levesque M., Parent A. (2002). Newly generated neurons in the amygdala and adjoining cortex of adult primates. Proc. Natl. Acad. Sci. USA.

[9] Gould E., Reeves A.J., Graziano M.S., Gross C.G. (1999). Neurogenesis in the neocortex of adult primates. Science.

[10] Carlén M., Cassidy R.M., Brismar H., Smith G.A., Enquist L.W., Frisén J. (2002). Functional integration of adult-born neurons. Curr. Biol. CB.

[11] Bi B., Salmaso N., Komitova M., Simonini M.V., Silbereis J., Cheng E., Kim J., Luft S., Ment L.R., Horvath T.L. (2011). Cortical glial fibrillary acidic protein-positive cells generate neurons after perinatal hypoxic injury. J. Neurosci. Off. J. Soc. Neurosci..

[12] Gotz M., Sirko S., Beckers J., Irmler M. (2015). Reactive astrocytes as neural stem or progenitor cells: In vivo lineage, In vitro potential, and Genome-wide expression analysis. Glia.

[13] Walther C., Gruss P. (1991). Pax-6, a murine paired box gene, is expressed in the developing CNS. Development.

[14] Hack M.A., Sugimori M., Lundberg C., Nakafuku M., Götz M. (2004). Regionalization and fate specification in neurospheres: The role of Olig2 and Pax6. Mol. Cell. Neurosci..

[15] Simpson T.I., Price D.J. (2002). Pax6; a pleiotropic player in development. BioEssays News Rev. Mol. Cell. Dev. Biol..

[16] Ton C.C., Miwa H., Saunders G.F. (1992). Small eye (Sey): Cloning and characterization of the murine homolog of the human aniridia gene. Genomics.

[17] Sansom S.N., Griffiths D.S., Faedo A., Kleinjan D.J., Ruan Y., Smith J., van Heyningen V., Rubenstein J.L., Livesey F.J. (2009). The level of the transcription factor Pax6 is essential for controlling the balance between neural stem cell self-renewal and neurogenesis. PLoS Genet..

[18] Miao Y., Xia Q., Hou Z., Zheng Y., Pan H., Zhu S. (2007). Ghrelin protects cortical neuron against focal ischemia/reperfusion in rats. Biochem. Biophys. Res. Commun..

[19] Liu J., Chen M., Dong R., Sun C., Li S., Zhu S. (2019). Ghrelin Promotes Cortical Neurites Growth in Late Stage After Oxygen-Glucose Deprivation/Reperfusion Injury. J. Mol. Neurosci. MN.

[20] Kilkenny C., Browne W.J., Cuthill I.C., Emerson M., Altman D.G. (2010). Improving bioscience research reporting: The ARRIVE guidelines for reporting animal research. PLoS Biol..

[21] Doeppner T.R., Herz J., Bähr M., Tonchev A.B., Stoykova A. (2019). Zbtb20 Regulates Developmental Neurogenesis in the Olfactory Bulb and Gliogenesis After Adult Brain Injury. Mol. Neurobiol..

[22] Romijn H.J., van Huizen F., Wolters P.S. (1984). Towards an improved serum-free, chemically defined medium for long-term culturing of cerebral cortex tissue. Neurosci. Biobehav. Rev..

[23] Matthiesen S., Jahnke R., Knittler M.R. (2021). A Straightforward Hypoxic Cell Culture Method Suitable for Standard Incubators. Methods Protoc..

[24] Cowley M.A., Smith R.G., Diano S., Tschöp M., Pronchuk N., Grove K.L., Strasburger C.J., Bidlingmaier M., Esterman M., Heiman M.L. (2003). The distribution and mechanism of action of ghrelin in the CNS demonstrates a novel hypothalamic circuit regulating energy homeostasis. Neuron.

[25] Johansson I., Destefanis S., Aberg N.D., Aberg M.A., Blomgren K., Zhu C., Ghe C., Granata R., Ghigo E., Muccioli G. (2008). Proliferative and protective effects of growth hormone secretagogues on adult rat hippocampal progenitor cells. Endocrinology.

[26] Diano S., Farr S.A., Benoit S.C., McNay E.C., da Silva I., Horvath B., Gaskin F.S., Nonaka N., Jaeger L.B., Banks W.A. (2006). Ghrelin controls hippocampal spine synapse density and memory performance. Nat. Neurosci..

[27] Stoyanova I.I., le Feber J. (2014). Ghrelin accelerates synapse formation and activity development in cultured cortical networks. BMC Neurosci..

[28] Kosugi S., Hasebe M., Tomita M., Yanagawa H. (2009). Systematic identification of cell cycle-dependent yeast nucleocytoplasmic shuttling proteins by prediction of composite motifs. Proc. Natl. Acad. Sci. USA.

[29] Moon M., Kim S., Hwang L., Park S. (2009). Ghrelin regulates hippocampal neurogenesis in adult mice. Endocr. J..

[30] Inoue Y., Nakahara K., Kangawa K., Murakami N. (2010). Transitional change in rat fetal cell proliferation in response to ghrelin and des-acyl ghrelin during the last stage of pregnancy. Biochem. Biophys. Res. Commun..

[31] Kojima M., Hosoda H., Date Y., Nakazato M., Matsuo H., Kangawa K. (1999). Ghrelin is a growth-hormone-releasing acylated peptide from stomach. Nature.

[32] Bednarek M.A., Feighner S.D., Pong S.S., McKee K.K., Hreniuk D.L., Silva M.V., Warren V.A., Howard A.D., Van Der Ploeg L.H., Heck J.V. (2000). Structure-function studies on the new growth hormone-releasing peptide, ghrelin: Minimal sequence of ghrelin necessary for activation of growth hormone secretagogue receptor 1a. J. Med. Chem..

[33] Guan X.M., Yu H., Palyha O.C., McKee K.K., Feighner S.D., Sirinathsinghji D.J., Smith R.G., Van der Ploeg L.H., Howard A.D. (1997). Distribution of mRNA encoding the growth hormone secretagogue receptor in brain and peripheral tissues. Brain Res. Mol. Brain Res..

[34] Muccioli G., Ghè C., Ghigo M.C., Papotti M., Arvat E., Boghen M.F., Nilsson M.H., Deghenghi R., Ong H., Ghigo E. (1998). Specific receptors for synthetic GH secretagogues in the human brain and pituitary gland. J. Endocrinol..

[35] Mitchell V., Bouret S., Beauvillain J.C., Schilling A., Perret M., Kordon C., Epelbaum J. (2001). Comparative distribution of mRNA encoding the growth hormone secretagogue-receptor (GHS-R) in Microcebus murinus (Primate, lemurian) and rat forebrain and pituitary. J. Comp. Neurol..

[36] Zigman J.M., Jones J.E., Lee C.E., Saper C.B., Elmquist J.K. (2006). Expression of ghrelin receptor mRNA in the rat and the mouse brain. J. Comp. Neurol..

[37] Andrews Z.B. (2011). The extra-hypothalamic actions of ghrelin on neuronal function. Trends Neurosci..

[38] Bron R., Yin L., Russo D., Furness J.B. (2013). Expression of the ghrelin receptor gene in neurons of the medulla oblongata of the rat. J. Comp. Neurol..

[39] Cabral A., Fernandez G., Tolosa M.J., Rey Moggia Á., Calfa G., De Francesco P.N., Perello M. (2020). Fasting induces remodeling of the orexigenic projections from the arcuate nucleus to the hypothalamic paraventricular nucleus, in a growth hormone secretagogue receptor-dependent manner. Mol. Metab..

[40] Stoyanova I., Lutz D. (2021). Ghrelin-Mediated Regeneration and Plasticity After Nervous System Injury. Front. Cell Dev. Biol..

[41] Huang J., Liu W., Doycheva D.M., Gamdzyk M., Lu W., Tang J., Zhang J.H. (2019). Ghrelin attenuates oxidative stress and neuronal apoptosis via GHSR-1α/AMPK/Sirt1/PGC-1α/UCP2 pathway in a rat model of neonatal HIE. Free Radic. Biol. Med..

[42] Stoyanova I.I., le Feber J., Rutten W.L. (2013). Ghrelin stimulates synaptic formation in cultured cortical networks in a dose-dependent manner. Regul. Pept..

[43] Cecarini V., Bonfili L., Cuccioloni M., Keller J.N., Bruce-Keller A.J., Eleuteri A.M. (2016). Effects of Ghrelin on the Proteolytic Pathways of Alzheimer’s Disease Neuronal Cells. Mol. Neurobiol..

[44] Fry M., Ferguson A.V. (2009). Ghrelin modulates electrical activity of area postrema neurons. Am. J. Physiol. Regul. Integr. Comp. Physiol..

[45] Yanagida H., Morita T., Kim J., Yoshida K., Nakajima K., Oomura Y., Wayner M.J., Sasaki K. (2008). Effects of ghrelin on neuronal activity in the ventromedial nucleus of the hypothalamus in infantile rats: An in vitro study. Peptides.

[46] Stoyanova I.I., Hofmeijer J., van Putten M., le Feber J. (2016). Acyl Ghrelin Improves Synapse Recovery in an In Vitro Model of Postanoxic Encephalopathy. Mol. Neurobiol..

[47] Suda Y., Kuzumaki N., Sone T., Narita M., Tanaka K., Hamada Y., Iwasawa C., Shibasaki M., Maekawa A., Matsuo M. (2018). Down-regulation of ghrelin receptors on dopaminergic neurons in the substantia nigra contributes to Parkinson’s disease-like motor dysfunction. Mol. Brain.

[48] Albarran-Zeckler R.G., Brantley A.F., Smith R.G. (2012). Growth hormone secretagogue receptor (GHS-R1a) knockout mice exhibit improved spatial memory and deficits in contextual memory. Behav. Brain Res..

[49] Erdmann J., Töpsch R., Lippl F., Gussmann P., Schusdziarra V. (2004). Postprandial response of plasma ghrelin levels to various test meals in relation to food intake, plasma insulin, and glucose. J. Clin. Endocrinol. Metab..

[50] Kronenberg G., Gertz K., Cheung G., Buffo A., Kettenmann H., Götz M., Endres M. (2010). Modulation of fate determinants Olig2 and Pax6 in resident glia evokes spiking neuroblasts in a model of mild brain ischemia. Stroke.

[51] Grande A., Sumiyoshi K., López-Juárez A., Howard J., Sakthivel B., Aronow B., Campbell K., Nakafuku M. (2013). Environmental impact on direct neuronal reprogramming in vivo in the adult brain. Nat. Commun..

[52] Torper O., Pfisterer U., Wolf D.A., Pereira M., Lau S., Jakobsson J., Björklund A., Grealish S., Parmar M. (2013). Generation of induced neurons via direct conversion in vivo. Proc. Natl. Acad. Sci. USA.

[53] Shihabuddin L.S., Horner P.J., Ray J., Gage F.H. (2000). Adult spinal cord stem cells generate neurons after transplantation in the adult dentate gyrus. J. Neurosci..

[54] Fong S.P., Tsang K.S., Chan A.B., Lu G., Poon W.S., Li K., Baum L.W., Ng H.K. (2007). Trophism of neural progenitor cells to embryonic stem cells: Neural induction and transplantation in a mouse ischemic stroke model. J. Neurosci. Res..

[55] Nacher J., Varea E., Blasco-Ibañez J.M., Castillo-Gomez E., Crespo C., Martinez-Guijarro F.J., McEwen B.S. (2005). Expression of the transcription factor Pax 6 in the adult rat dentate gyrus. J. Neurosci. Res..

[56] Englund C., Fink A., Lau C., Pham D., Daza R.A., Bulfone A., Kowalczyk T., Hevner R.F. (2005). Pax6, Tbr2, and Tbr1 are expressed sequentially by radial glia, intermediate progenitor cells, and postmitotic neurons in developing neocortex. J. Neurosci. Off. J. Soc. Neurosci..

[57] Warren N., Caric D., Pratt T., Clausen J.A., Asavaritikrai P., Mason J.O., Hill R.E., Price D.J. (1999). The transcription factor, Pax6, is required for cell proliferation and differentiation in the developing cerebral cortex. Cereb. Cortex.

[58] Walcher T., Xie Q., Sun J., Irmler M., Beckers J., Öztürk T., Niessing D., Stoykova A., Cvekl A., Ninkovic J. (2013). Functional dissection of the paired domain of Pax6 reveals molecular mechanisms of coordinating neurogenesis and proliferation. Development.

[59] Estivill-Torrus G., Pearson H., van Heyningen V., Price D.J., Rashbass P. (2002). Pax6 is required to regulate the cell cycle and the rate of progression from symmetrical to asymmetrical division in mammalian cortical progenitors. Development.

[60] Asami M., Pilz G.A., Ninkovic J., Godinho L., Schroeder T., Huttner W.B., Götz M. (2011). The role of Pax6 in regulating the orientation and mode of cell division of progenitors in the mouse cerebral cortex. Development.

[61] Ahmad Z., Rafeeq M., Collombat P., Mansouri A. (2015). Pax6 Inactivation in the Adult Pancreas Reveals Ghrelin as Endocrine Cell Maturation Marker. PLoS ONE.

[62] Haubst N., Berger J., Radjendirane V., Graw J., Favor J., Saunders G.F., Stoykova A., Götz M. (2004). Molecular dissection of Pax6 function: The specific roles of the paired domain and homeodomain in brain development. Development.

[63] Ninkovic J., Steiner-Mezzadri A., Jawerka M., Akinci U., Masserdotti G., Petricca S., Fischer J., von Holst A., Beckers J., Lie C.D. (2013). The BAF complex interacts with Pax6 in adult neural progenitors to establish a neurogenic cross-regulatory transcriptional network. Cell Stem Cell.

[64] Agoston Z., Heine P., Brill M.S., Grebbin B.M., Hau A.C., Kallenborn-Gerhardt W., Schramm J., Götz M., Schulte D. (2014). Meis2 is a Pax6 co-factor in neurogenesis and dopaminergic periglomerular fate specification in the adult olfactory bulb. Development.

[65] Tuoc T.C., Boretius S., Sansom S.N., Pitulescu M.E., Frahm J., Livesey F.J., Stoykova A. (2013). Chromatin regulation by BAF170 controls cerebral cortical size and thickness. Dev. Cell.

[66] Mussa Z., Tome-Garcia J., Jiang Y., Akbarian S., Tsankova N.M. (2021). Isolation of Adult Human Astrocyte Populations from Fresh-frozen Cortex using Fluorescence-Activated Nuclei Sorting. J. Vis. Exp. JoVE.

[67] Falcone C., Penna E., Hong T., Tarantal A.F., Hof P.R., Hopkins W.D., Sherwood C.C., Noctor S.C., Martínez-Cerdeño V. (2021). Cortical Interlaminar Astrocytes Are Generated Prenatally, Mature Postnatally, and Express Unique Markers in Human and Nonhuman Primates. Cereb. Cortex.

[68] Bruno S., Darzynkiewicz Z. (1992). Cell cycle dependent expression and stability of the nuclear protein detected by Ki-67 antibody in HL-60 cells. Cell Prolif..

[69] Scholzen T., Gerdes J. (2000). The Ki-67 protein: From the known and the unknown. J. Cell. Physiol..

[70] Gerdes J., Lemke H., Baisch H., Wacker H.H., Schwab U., Stein H. (1984). Cell cycle analysis of a cell proliferation-associated human nuclear antigen defined by the monoclonal antibody Ki-67. J. Immunol..

[71] Gerdes J., Schwab U., Lemke H., Stein H. (1983). Production of a mouse monoclonal antibody reactive with a human nuclear antigen associated with cell proliferation. Int. J. Cancer.

[72] Sun X., Kaufman P.D. (2018). Ki-67: More than a proliferation marker. Chromosoma.

[73] Booth D.G., Beckett A.J., Molina O., Samejima I., Masumoto H., Kouprina N., Larionov V., Prior I.A., Earnshaw W.C. (2016). 3D-CLEM Reveals that a Major Portion of Mitotic Chromosomes Is Not Chromatin. Mol. Cell.

[74] Cuylen S., Blaukopf C., Politi A.Z., Müller-Reichert T., Neumann B., Poser I., Ellenberg J., Hyman A.A., Gerlich D.W. (2016). Ki-67 acts as a biological surfactant to disperse mitotic chromosomes. Nature.

[75] Booth D.G., Takagi M., Sanchez-Pulido L., Petfalski E., Vargiu G., Samejima K., Imamoto N., Ponting C.P., Tollervey D., Earnshaw W.C. (2014). Ki-67 is a PP1-interacting protein that organises the mitotic chromosome periphery. eLife.

[76] du Manoir S., Guillaud P., Camus E., Seigneurin D., Brugal G. (1991). Ki-67 labeling in postmitotic cells defines different Ki-67 pathways within the 2c compartment. Cytometry.

[77] Starborg M., Gell K., Brundell E., Höög C. (1996). The murine Ki-67 cell proliferation antigen accumulates in the nucleolar and heterochromatic regions of interphase cells and at the periphery of the mitotic chromosomes in a process essential for cell cycle progression. J. Cell Sci..

[78] Moon M., Cha M.Y., Mook-Jung I. (2014). Impaired hippocampal neurogenesis and its enhancement with ghrelin in 5XFAD mice. J. Alzheimers Dis. JAD.

[79] Li E., Kim Y., Kim S., Sato T., Kojima M., Park S. (2014). Ghrelin stimulates proliferation, migration and differentiation of neural progenitors from the subventricular zone in the adult mice. Exp. Neurol..

[80] Chung H., Li E., Kim Y., Kim S., Park S. (2013). Multiple signaling pathways mediate ghrelin-induced proliferation of hippocampal neural stem cells. J. Endocrinol..

[81] Belayev L., Hong S.H., Menghani H., Marcell S.J., Obenaus A., Freitas R.S., Khoutorova L., Balaszczuk V., Jun B., Oriá R.B. (2018). Docosanoids Promote Neurogenesis and Angiogenesis, Blood-Brain Barrier Integrity, Penumbra Protection, and Neurobehavioral Recovery After Experimental Ischemic Stroke. Mol. Neurobiol..

[82] Heddleston J.M., Li Z., McLendon R.E., Hjelmeland A.B., Rich J.N. (2009). The hypoxic microenvironment maintains glioblastoma stem cells and promotes reprogramming towards a cancer stem cell phenotype. Cell Cycle.

[83] Kolenda J., Jensen S.S., Aaberg-Jessen C., Christensen K., Andersen C., Brünner N., Kristensen B.W. (2011). Effects of hypoxia on expression of a panel of stem cell and chemoresistance markers in glioblastoma-derived spheroids. J. Neuro-Oncol..

[84] Tonchev A.B., Yamashima T., Sawamoto K., Okano H. (2005). Enhanced proliferation of progenitor cells in the subventricular zone and limited neuronal production in the striatum and neocortex of adult macaque monkeys after global cerebral ischemia. J. Neurosci. Res..

[85] Tonchev A.B., Yamashima T., Zhao L., Okano H.J., Okano H. (2003). Proliferation of neural and neuronal progenitors after global brain ischemia in young adult macaque monkeys. Mol. Cell. Neurosci..

[86] Macas J., Nern C., Plate K.H., Momma S. (2006). Increased generation of neuronal progenitors after ischemic injury in the aged adult human forebrain. J. Neurosci. Off. J. Soc. Neurosci..

[87] Chiasson B.J., Tropepe V., Morshead C.M., van der Kooy D. (1999). Adult mammalian forebrain ependymal and subependymal cells demonstrate proliferative potential, but only subependymal cells have neural stem cell characteristics. J. Neurosci. Off. J. Soc. Neurosci..

[88] Nunes M.C., Roy N.S., Keyoung H.M., Goodman R.R., McKhann G., Jiang L., Kang J., Nedergaard M., Goldman S.A. (2003). Identification and isolation of multipotential neural progenitor cells from the subcortical white matter of the adult human brain. Nat. Med..

[89] Sanai N., Tramontin A.D., Quiñones-Hinojosa A., Barbaro N.M., Gupta N., Kunwar S., Lawton M.T., McDermott M.W., Parsa A.T., Manuel-García Verdugo J. (2004). Unique astrocyte ribbon in adult human brain contains neural stem cells but lacks chain migration. Nature.

[90] Ortega J.A., Sirois C.L., Memi F., Glidden N., Zecevic N. (2017). Oxygen Levels Regulate the Development of Human Cortical Radial Glia Cells. Cereb. Cortex.

[91] le Feber J., Erkamp N., van Putten M. (2017). Loss and recovery of functional connectivity in cultured cortical networks exposed to hypoxia. J. Neurophysiol..

[92] Sugawara T., Lewén A., Noshita N., Gasche Y., Chan P.H. (2002). Effects of global ischemia duration on neuronal, astroglial, oligodendroglial, and microglial reactions in the vulnerable hippocampal CA1 subregion in rats. J. Neurotrauma.

[93] Unal-Cevik I., Kilinç M., Gürsoy-Ozdemir Y., Gurer G., Dalkara T. (2004). Loss of NeuN immunoreactivity after cerebral ischemia does not indicate neuronal cell loss: A cautionary note. Brain Res..

[94] Mullen R.J., Buck C.R., Smith A.M. (1992). NeuN, a neuronal specific nuclear protein in vertebrates. Development.

[95] McPhail L.T., McBride C.B., McGraw J., Steeves J.D., Tetzlaff W. (2004). Axotomy abolishes NeuN expression in facial but not rubrospinal neurons. Exp. Neurol..

[96] Hossmann K.A. (1993). Disturbances of cerebral protein synthesis and ischemic cell death. Prog. Brain Res..

[97] Duan W., Zhang Y.P., Hou Z., Huang C., Zhu H., Zhang C.Q., Yin Q. (2016). Novel Insights into NeuN: From Neuronal Marker to Splicing Regulator. Mol. Neurobiol..

[98] Barbosa-Morais N.L., Irimia M., Pan Q., Xiong H.Y., Gueroussov S., Lee L.J., Slobodeniuc V., Kutter C., Watt S., Colak R. (2012). The evolutionary landscape of alternative splicing in vertebrate species. Science.

[99] Kim K.K., Nam J., Mukouyama Y.S., Kawamoto S. (2013). Rbfox3-regulated alternative splicing of Numb promotes neuronal differentiation during development. J. Cell Biol..

[100] Lal D., Reinthaler E.M., Altmüller J., Toliat M.R., Thiele H., Nürnberg P., Lerche H., Hahn A., Møller R.S., Muhle H. (2013). RBFOX1 and RBFOX3 mutations in rolandic epilepsy. PLoS ONE.

[101] Maxeiner S., Glassmann A., Kao H.T., Schilling K. (2014). The molecular basis of the specificity and cross-reactivity of the NeuN epitope of the neuron-specific splicing regulator, Rbfox3. Histochem. Cell Biol..

