# Supplementary material for: Ghrelin Regulates Expression of the Transcription Factor Pax6 in Hypoxic Brain Progenitor Cells and Neurons
Source: Cells. 2022 Feb 23;11(5):782. doi: 10.3390/cells11050782 (PMC8909042; doi:10.3390/cells11050782)
Supplement: Supplementary file 1 [file cells-11-00782-s001.zip › cells-1550192-supplementary.pdf]

*Research Article (**Supplementary Material**)*

Ghrelin Regulates Expression of the Transcription Factor Pax6 in Hypoxic Brain Progenitor Cells and Neurons

Irina I. Stoyanova [1,*], Andrii Klymenko [2], Jeannette Willms [2], Thorsten R. Döppner [3,4], Anton B. Tonchev [1] and David Lutz [2,*]

1 Department of Anatomy and Cell Biology, Faculty of Medicine, and Research Institute of the Medical
University, 9002 Varna, Bulgaria; anton.tonchev@mu-varna.bg

2 Department of Neuroanatomy and Molecular Brain Research, Ruhr University Bochum, 44801 Bochum, Germany; andrii.klymenko@rub.de (A.K.); Jeannette.Willms@ruhr-uni-bochum.de (J.W.)

3 Department of Neurology, University Medical Center Goettingen, 37075 Goettingen, Germany;
thorsten.doeppner@med.uni-goettingen.de

4 Research Institute for Health Sciences and Technologies (SABITA), Medipol University, 34810 Istanbul, Turkey

* Correspondence: stoyanovai@yahoo.co.uk (I.I.S.); david.lutz@rub.de (D.L.)

Control immunostaining for the GHSR1 antibody

To test the specificity of the GHSR1 antibody, we conducted two control staining procedures as follows. Prior to staining, we incubated the murine primary GHSR1 antibody with murine pituitary/hypothalamus tissue homogenate to bind the antibody with detergent-solubilised GHSR1. In particular, tissue of pituitary gland and hypothalamus from an 11–month–old male mouse was freshly isolated and homogenised in RIPA buffer (150 mM sodium chloride, 50 mM tris-HCl, 1% Nonidet P–40, 0.5% sodium deoxycholate, pH 8.0). The homogenate was then centrifuged at 13,000 rpm (room temperature) for 10 min und the supernatant was used for further incubation steps. Approximately 120 µg of the supernatant protein were mixed with 4 ng of the GHRS1 antibody at room temperature for 30 min. This mixture was applied to fixed cerebral cortex cells at 4 °C for 10 hours, followed by the standard steps of immunocytochemistry as described above (Supplementary Figure S1A). For the second control staining procedure, we applied only the Alexa 594–conjugated secondary antibody (without the primary GHSR1 antibody) to the cells (using a dilution of 1:1000) for 2 hours (Supplementary Figure S1B). In both cases, the GHSR1 fluorescence signal was barely detectable with the set-up of the spinning disk microscope as described in the imaging acquisition section (Supplementary Figures 1A,B).

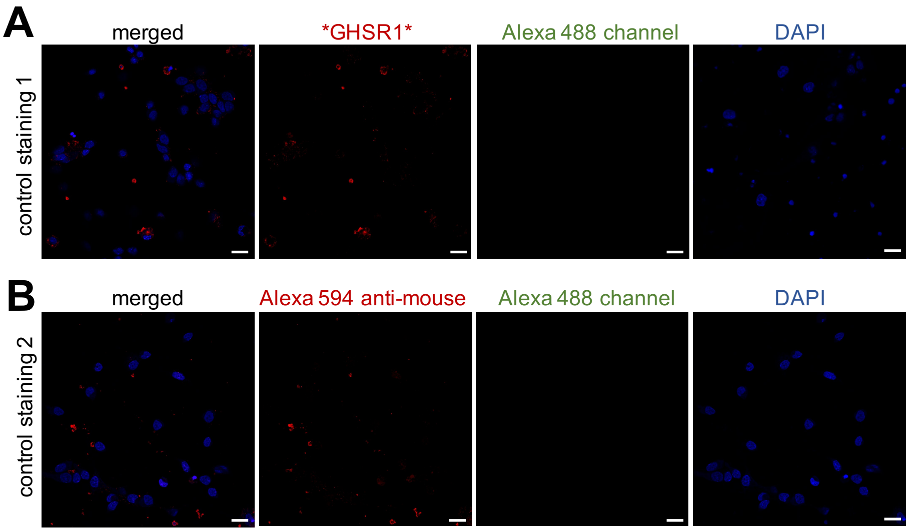

Supplementary Figure S1. Control immunostaining experiments for the GHSR1 antibody referring to Figure 2 of the main text. (**A**) Control immunostaining with the GHSR1 antibody (red) that had been incubated with detergent-solubilised homogenate of the pituitary gland/hypothalamus tissue (*GHSR1*) prior to application to fixed cerebral cortex cells. (**B**) Immunostaining with the anti-mouse secondary antibody (Alexa 594, red), without using the primary GHSR1 antibody. (**A,B**) The channels for the Alexa 488 cannel (green) are shown. DAPI (blue) was used to stain the nuclei. Scale bar, 30 µm.

Uncropped images of the dot blots

Cerebral cortex slices were homogenised as described in Methods. For each condition, 20 µg total protein per dot were analysed with the murine antibody recognising GHSR1 (cat.# sc-374515, RRID:AB_10987651, Santa Cruz Biotechnology, Dallay, TX, USA, dilution 1:1000, incubation overnight) or a mouse β-actin antibody (cat.# A5441, RRID:AB_476744, Sigma-Aldrich, St. Louis, MO, USA, dilution 1:10,000, incubation for 1 hour). The duration of the exposure for the GHSR1 and β-actin signal were 10 s and 20 s, respectively (Supplementary Figure S2). The membranes were finally stained with Ponceau S solution for 4 min, washed in distilled water for 15 min and air-dried (Supplementary Figure S2).

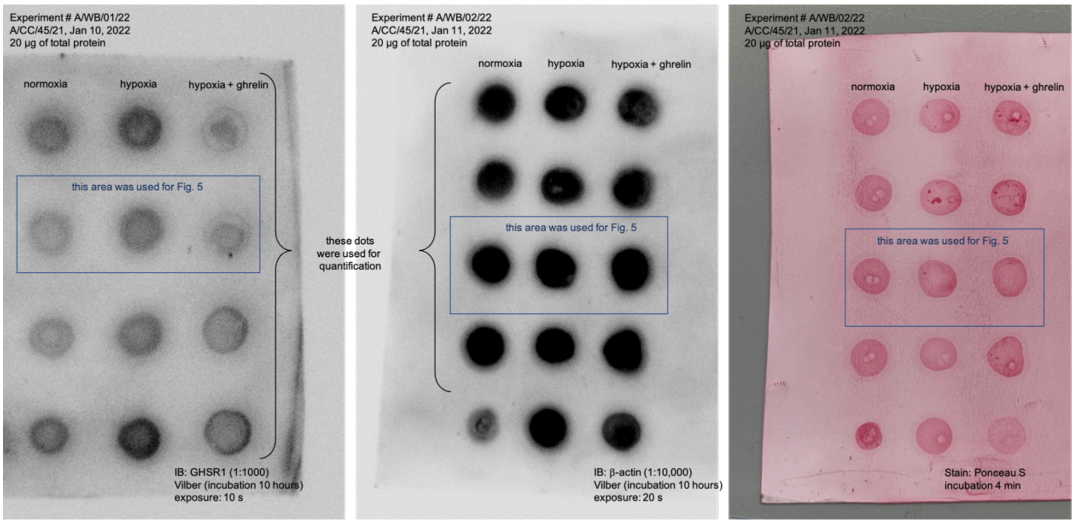

Supplementary Figure S2. Uncropped dot blot images referring to Figure 5 of the main text. Areas shown in the main figures and used for quantification are indicated.
